# Longitudinal network-based brain grey matter MRI measures are clinically relevant and sensitive to treatment effects in multiple sclerosis

**DOI:** 10.1093/braincomms/fcae234

**Published:** 2024-07-29

**Authors:** Elisa Colato, Jonathan Stutters, Sridar Narayanan, Douglas L Arnold, Jeremy Chataway, Claudia A M Gandini Wheeler-Kingshott, Frederik Barkhof, Olga Ciccarelli, Arman Eshaghi, Declan T Chard

**Affiliations:** Queen Square Multiple Sclerosis Centre, Department of Neuroinflammation, University College London (UCL) Queen Square Institute of Neurology, Faculty of Brain Sciences, University College London, London, WC1N 3BG, UK; Queen Square Multiple Sclerosis Centre, Department of Neuroinflammation, University College London (UCL) Queen Square Institute of Neurology, Faculty of Brain Sciences, University College London, London, WC1N 3BG, UK; McConnell Brain Imaging Centre, Montreal Neurological Institute, McGill University, Montreal, Quebec, H3A 2B4, Canada; McConnell Brain Imaging Centre, Montreal Neurological Institute, McGill University, Montreal, Quebec, H3A 2B4, Canada; Queen Square Multiple Sclerosis Centre, Department of Neuroinflammation, University College London (UCL) Queen Square Institute of Neurology, Faculty of Brain Sciences, University College London, London, WC1N 3BG, UK; National Institute for Health Research (NIHR) University College London Hospitals (UCLH) Biomedical Research Centre (BRC), London, W1T 7DN, UK; Queen Square Multiple Sclerosis Centre, Department of Neuroinflammation, University College London (UCL) Queen Square Institute of Neurology, Faculty of Brain Sciences, University College London, London, WC1N 3BG, UK; Brain Connectivity Centre, Istituto di Ricovero e Cura a Carattere Scientifico (IRCCS) Mondino Foundation, Pavia, 27100, Italy; Department of Brain and Behavioural Sciences, University of Pavia, Pavia, 27100, Italy; Queen Square Multiple Sclerosis Centre, Department of Neuroinflammation, University College London (UCL) Queen Square Institute of Neurology, Faculty of Brain Sciences, University College London, London, WC1N 3BG, UK; National Institute for Health Research (NIHR) University College London Hospitals (UCLH) Biomedical Research Centre (BRC), London, W1T 7DN, UK; Department of Radiology and Nuclear Medicine, Vrije Universiteit (VU) Medical Centre, Amsterdam, 1081 HZ, The Netherlands; Centre for Medical Image Computing (CMIC), Department of Medical Physics and Biomedical Engineering, University College London, London, WC1V 6LJ, UK; Queen Square Multiple Sclerosis Centre, Department of Neuroinflammation, University College London (UCL) Queen Square Institute of Neurology, Faculty of Brain Sciences, University College London, London, WC1N 3BG, UK; National Institute for Health Research (NIHR) University College London Hospitals (UCLH) Biomedical Research Centre (BRC), London, W1T 7DN, UK; Queen Square Multiple Sclerosis Centre, Department of Neuroinflammation, University College London (UCL) Queen Square Institute of Neurology, Faculty of Brain Sciences, University College London, London, WC1N 3BG, UK; Centre for Medical Image Computing (CMIC), Department of Computer Science, University College London, London, WC1V 6LJ, UK; Queen Square Multiple Sclerosis Centre, Department of Neuroinflammation, University College London (UCL) Queen Square Institute of Neurology, Faculty of Brain Sciences, University College London, London, WC1N 3BG, UK; National Institute for Health Research (NIHR) University College London Hospitals (UCLH) Biomedical Research Centre (BRC), London, W1T 7DN, UK

**Keywords:** multiple sclerosis, independent component analysis, grey matter, disability, treatment effects

## Abstract

In multiple sclerosis clinical trials, MRI outcome measures are typically extracted at a whole-brain level, but pathology is not homogeneous across the brain and so whole-brain measures may overlook regional treatment effects. Data-driven methods, such as independent component analysis, have shown promise in identifying regional disease effects but can only be computed at a group level and cannot be applied prospectively. The aim of this work was to develop a technique to extract longitudinal independent component analysis network-based measures of co-varying grey matter volumes, derived from T_1_-weighted volumetric MRI, in individual study participants, and assess their association with disability progression and treatment effects in clinical trials. We used longitudinal MRI and clinical data from 5089 participants (22 045 visits) with multiple sclerosis from eight clinical trials. We included people with relapsing–remitting, primary and secondary progressive multiple sclerosis. We used data from five negative clinical trials (2764 participants, 13 222 visits) to extract the independent component analysis-based measures. We then trained and cross-validated a least absolute shrinkage and selection operator regression model (which can be applied prospectively to previously unseen data) to predict the independent component analysis measures from the same regional MRI volume measures and applied it to data from three positive clinical trials (2325 participants, 8823 visits). We used nested mixed-effect models to determine how networks differ across multiple sclerosis phenotypes are associated with disability progression and to test sensitivity to treatment effects. We found 17 consistent patterns of co-varying regional volumes. In the training cohort, volume loss was faster in four networks in people with secondary progressive compared with relapsing–remitting multiple sclerosis and three networks with primary progressive multiple sclerosis. Volume changes were faster in secondary compared with primary progressive multiple sclerosis in four networks. In the combined positive trials cohort, eight independent component analysis networks and whole-brain grey matter volume measures showed treatment effects, and the magnitude of treatment–placebo differences in the network-based measures was consistently greater than with whole-brain grey matter volume measures. Longitudinal network-based analysis of grey matter volume changes is feasible using clinical trial data, showing differences cross-sectionally and longitudinally between multiple sclerosis phenotypes, associated with disability progression, and treatment effects. Future work is required to understand the pathological mechanisms underlying these regional changes.

## Teaser

Colato *et al*. present a new network-based measure of brain atrophy using longitudinal MRI that could be used prospectively in multiple sclerosis clinical trials and found networks of co-varying grey matter volumes that differed across clinical phenotypes, were associated with disability progression and showed treatment effects.

## Graphical Abstract

**Graphical abstract:**
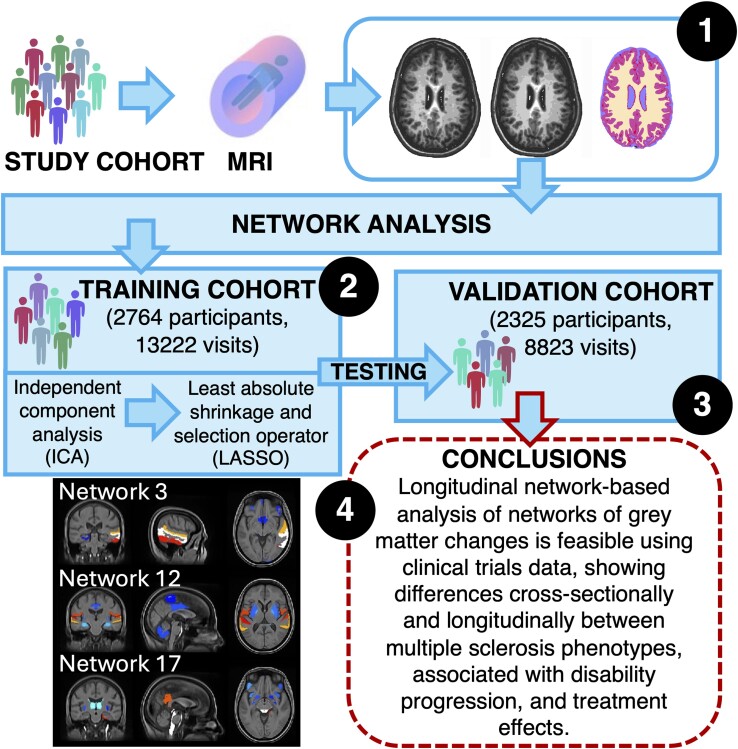

## Introduction

In multiple sclerosis, the main cause of irreversible progressive disability is thought to be neurodegeneration.^1,2^ While we have many treatments approved for relapsing–remitting (RR) multiple sclerosis, only a few have proven effective in progressive multiple sclerosis when there is still evidence of focal inflammatory activity. A key question for clinical trials is whether we can slow neurodegeneration that is not immediately linked with focal inflammation. Sensitive, and clinically relevant, measures of neurodegeneration are needed to address this question. Brain atrophy, measured with MRI, is the main *in vivo* marker of neurodegeneration used in clinical trials.^3-6^ However, it is usually measured at a whole-brain or whole grey matter (GM) level, but it has been shown that atrophy varies substantially between brain regions and that this differs between multiple sclerosis clinical phenotypes.^7^

Recently, independent component analysis (ICA) has been applied to brain MRI scans to unpick overlapping patterns, or networks, of GM atrophy.^8,9^ Bergsland *et al*.^8^ performed a longitudinal study using voxel-wise, network-based, ICA-based approach and found patterns including structurally or functionally related GM regions. It has been postulated that some brain networks (structurally or functionally connected brain regions) are more vulnerable to, or specifically targeted by, neurodegenerative pathologies, which leads to the complex patterns of regional brain atrophy that are seen.^10^ Furthermore, it has been suggested that network-based measures may better explain clinical outcomes than whole-brain measures and be more sensitive to clinically relevant changes in early-phase clinical trials.^11^ For example, we and others have shown that MRI measures derived from the motor network are more closely associated with Expanded Disability Status Scale (EDSS) than the whole-brain MRI measures.^12^ A crucial and unresolved question is whether the dynamics of structural network changes differ between multiple sclerosis phenotypes, and if so, how this relates to clinical progression. To our knowledge, no study has investigated the longitudinal evolution of GM volumes using a data-driven network-based approach.

While ICA is well established as a data-driven tool for network discovery, producing measures for individuals within a cohort, it must be computed simultaneously for the whole group. If there are new participants or time points, we need to re-estimate the whole ICA model using the new sample, which is computationally cumbersome and precludes its use prospectively in clinical trials, for example, to calculate interim outcome measures. Machine learning models are adept at mapping complex inputs and outputs at an individual level, and such models can be used to predict outcomes for new input data (supervised learning).^13,14^ The least absolute shrinkage and selection operator (Lasso) method is a machine learning regression method that can robustly model a large number of variables, which would not be possible with linear regression models.^13^ This approach is potentially well suited to map features extracted at a group level using ICA to measures that can prospectively assess individual patient network measures, without the need to refit ICA to the whole population of people when there is a new participant or time point. This in turn may be used to assess the effects of treatments on longitudinal network-based measures at an individual level.

In this study, we aimed to (i) develop a novel longitudinal measure of brain structural network changes that could be applied to previously unseen data, (ii) assess its potential utility by investigating relationships of the network-based measures with disability progression and multiple sclerosis phenotypes and (iii) determine if network measures can detect a treatment effect in clinical trials.

## Materials and methods

### Participants

We used longitudinal data from eight randomized, double-blind trials in RR multiple sclerosis, secondary progressive (SP) multiple sclerosis and primary progressive (PP) multiple sclerosis, collected under the auspices of the International Progressive Multiple Sclerosis Alliance and the Multiple Sclerosis-Secondary Progressive Multi-Arm Randomization Trial (MS-SMART) (Supplementary Table 1).^5,15-20^ We divided participants into training and application cohorts. The training sample included five clinical trials in RR multiple sclerosis, SP multiple sclerosis and PP multiple sclerosis that did not report significant treatment effects at a group level, and we used this cohort to train our models. In the application sample, we included two clinical trials in RR multiple sclerosis and one in PP multiple sclerosis that showed significant treatment effects. We divided the cohorts in this way to reduce the risks that treatment effects could distort model training and assess known treatment effects in the application cohort in greater detail.

### MRI processing

**Figure 1:**
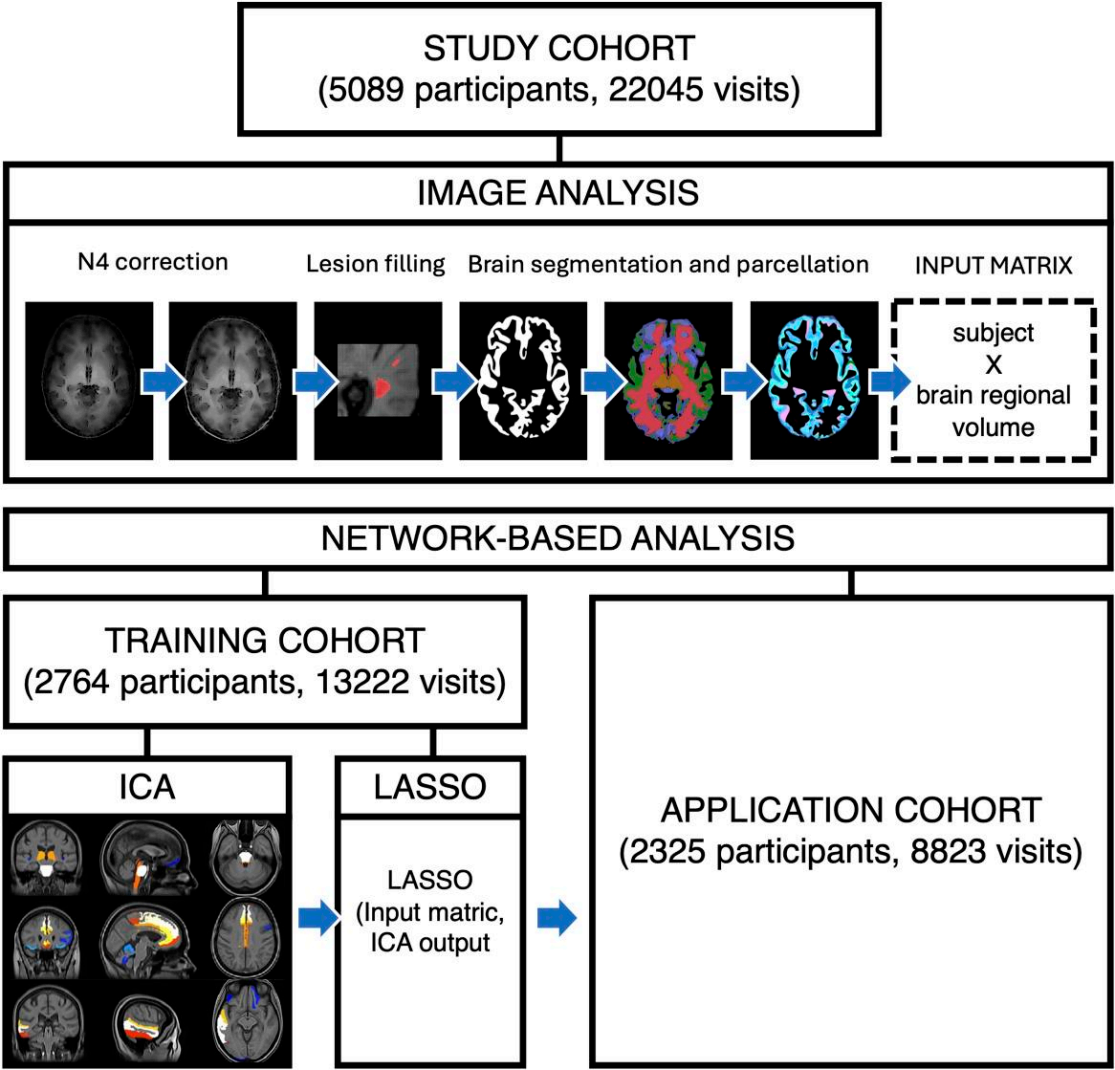
**Study design.** We processed all the available images as follows. We corrected T_1_ images for scanner inhomogeneities, performed lesion filling on T_1_ images, segmented, parcellated the brain and measured brain GM region volumes. We divided the study cohort into training and application cohorts based on whether data were obtained from a positive or negative clinical trial. We fitted a spatial ICA on the regional GM volume measures data from the training cohort. We fitted a machine learning model, using Lasso, to the ICA measures in the training cohort, and then used the fitted model to obtain network-based measures for each participant and time point in the application cohort.

We processed MRI data from the training and application cohorts as shown in Fig. 1 and detailed below. We used a pipeline similar to our previous work^21^ to process T_1_-weighted MRI scans and obtain GM volumes. We corrected T_1_-weighted scans for scanner inhomogeneities using N4 bias field correction toolbox.^22^ We used a convolutional–neural network-based model to automatically segment hyperintense lesions on T_2_-FLAIR scans^23^; the lesion masks were manually checked and used to fill T_1_-weighted hypointense lesions as per Prados *et al*.^24^ after registration with the Advanced Normalization Tools^25^; and the resulting images were segmented using Geodesic Information Flows version 3.0^26^ into GM and white matter, and CSF, across 125 brain GM regions based on the Neuromorphometric Atlas.^27^ We then obtained regional volumes by multiplying the sum of the probability of the segmented tissue voxels in each parcellated region with the voxel volume.

#### Network-based analysis

In the training cohort, we used spatial ICA to identify networks of co-varying GM regional volumes from GM regional volume measures. We applied the FastICA algorithm^28^ implemented in scikit-learn 0.23.1, allowing for up to 20 networks of co-varying brain regions volumes to be identified. We decided to include a larger number of components compared with previous studies using ICA (e.g. Bergsland *et al*.^8^ set the number of components to 8) to capture potentially interesting but less strong data patterns.^29^ Indeed, empirical studies have suggested that including a larger number of components, so potentially over-fitting rather than under-fitting, is better in terms of errors.^29^ From this, we obtained a network-based measure for each participant and each time point in the training cohort (Fig. 1).

In the training cohort, we then trained a Lasso regression model^30^ to obtain individual-level network-based measures for participants. We used the same regional GM volumes analysed with ICA as the input measures and the ICA network loadings as the target output measure. We split the training cohort into a training set (70% of the training cohort) and cross-validation set (the remaining 30% of the training cohort), to build and validate the Lasso model. We applied the fitted model to the application cohort to obtain individual-level network-based measures for each participant and each time point without the need to re-estimate the whole model parameters (Fig. 1).

While the training and application cohorts were deliberately separated based on treatment responses, as discussed earlier, we also repeated the ICA in the application cohort and performed pairwise spatial cross-correlations between components from the training and application cohorts to assess their stability. Specifically, we concatenated results from the training and application cohorts into two 4D images (each volume representing a distinct network) and used *fslcc*^31^ to determine spatial correlations between each volume of the two images. Additionally, a visual comparison was conducted to further validate the results.

We applied ComBat to harmonize GM network-based measures and whole-brain GM volume for the effect of different MRI vendors and sites using the neuroCombat package (version 1.0.13) in R.

### Statistical analysis

We used R (version 4.2) for statistical analysis. We corrected all analyses described below for multiple comparisons using false discovery rate using Benjamini–Hochberg procedure.

#### ICA and Lasso model characteristics

ICA components are not necessarily parallel to the measures underlying them (for an individual ICA, both positive and negative loading can represent lower GM volumes), and so we performed Pearson correlations between the ICA and Lasso loading for each network and the whole-brain GM volume measures. To aid readability, we inverted the sign of the loading for a network so that for the final results a negative loading equated to lower GM volumes. We used *R*^2^ to evaluate model fit. We assessed intra-class correlations between the ICA network measures and measures predicted through Lasso both for the training and the application cohort.

#### Clinical associations with GM network-based measures

To investigate differences in GM network measures at baseline and over time across multiple sclerosis phenotypes, in the training cohort (which included RR multiple sclerosis, SP multiple sclerosis and PP multiple sclerosis), we used mixed-effect models with Lasso loading factors of each network as the dependent variable. The independent variables included multiple sclerosis phenotype with SP multiple sclerosis as reference group, time (years from baseline) and the interaction between multiple sclerosis phenotype and time as fixed effects, baseline age, sex, disease duration, total intracranial volume and treatment as covariates. We used nested random effects models, in which visit was nested within each subject. We also repeated the analysis with RR multiple sclerosis as reference group. For context, we also repeated the analysis using whole-brain GM volume measures. To confirm that the Lasso model outputs effectively mirrored the ICA loading factors, we repeated these analyses using the loading factors of networks obtained using ICA.

To determine whether network loading factors could explain disability progression, in the training cohort, we built mixed-effect models with EDSS as the dependent variable. We selected eight GM networks involving brain regions (cerebellum, basal ganglia, premotor, associative, temporal and parietal cortices, pons and brainstem) known to be impaired and associated with disability in multiple sclerosis (i.e. Networks 2, 3, 11, 12, 13, 14, 18 and 19).^7,21,32-35^ These models included GM network loading factor (as *z*-scores), time and the interaction between them: baseline age, sex, disease duration, total intracranial volume and treatment as independent, fixed-effect variables. Again, we repeated these analyses using both the Lasso model outputs and the source ICA-based loading factors to determine if they were consistent.

To determine the independent predictive ability of network-based measures, in the training cohort, we undertook a stepwise linear regression starting with all the network-based measures that were significant in the mixed-effect models, along with whole-brain GM volumes, and correcting for baseline age, sex, disease duration, treatment line, total intracranial volume and number of visits. The stepwise models were run separately for baseline EDSS scores and longitudinal EDSS changes.

#### Detecting treatment effects

In the application cohort, to determine whether network-based measures could detect treatment effects, we built a mixed-effect model with the interaction between time and treatment arm, baseline age, sex and total intracranial volume as fixed-effect variables and network measures as outcomes.

We repeated these analyses using Lasso- and ICA-network measures to determine their consistency. For all the significant measures, we estimated the difference in each measure between the first and last visits, computed the effect size by taking the difference between the placebo and treated and ran a sample size calculation (*P* = 0.05, power = 0.80, *pwr* package).

## Results

### Demographics

We included clinical and MRI data for 5089 participants with multiple sclerosis (22 045 visits). The training cohort included data from 2764 participants (1060 RR multiple sclerosis, 1282 SP multiple sclerosis and 422 PP multiple sclerosis), 13 222 visits, with a mean follow-up of 3.4 (SD = 2.5) years. The application cohort comprised data from 2325 participants (1624 RR multiple sclerosis and 701 PP multiple sclerosis), 8823 visits, with a mean follow-up of 2.1 (0.7) years. Table 1 shows the demographic and clinical characteristics of cohorts.

**Table 1:** Demographics

	Entire cohort (*N* = 5089)	Training cohort (*N* = 2764; 54%)				Application cohort (*N* = 2325; 46%)		
		Whole	RR multiple sclerosis (*N* = 1060, 39%)	SP multiple sclerosis (*N* = 1282, 46%)	PP multiple sclerosis (*N* = 422, 15%)	Whole	RR multiple sclerosis (*N* = 1624, 70%)	PP multiple sclerosis (*N* = 701, 30%)
Age (years)	42.4 (10.4)	45.0 (10.5)	37.5 (9.1)	49.5 (8.5)	49.9 (8.9)	39.4 (9.5)	37.1 (9.2)	44.6 (8.0)
Sex (M/F)	1873/3216	966/1798	293/767	464/818	209/213	907/1418	554/1070	353/348
Disease duration (years)	6.4 (7.1)	8.71(8.1)	4.9 (5.2)	13.5 (8.4)	4.0 (4.3)	3.6 (4.5)	4.0 (4.9)	2.8 (3.1)
Total *N* visits	22 045	13 222	5197	6183	1842	8823	6072	2751
*N* visits	4.3 (1.9)	4.8 (2.3)	4.9 (2.7)	4.8 (2.3)	4.4 (1.7)	3.8 (0.8)	3.7 (0.8)	3.9 (0.8)
Follow-up (years)	2.8 (2.0)	3.4 (2.5)	5.3 (3.1)	2.2 (0.9)	2.2 (0.6)	2.1 (0.7)	2.2 (0.8)	2.1 (0.6)
Baseline EDSS^a^	4.0 [2.5–5.5]	4.5 [3.0–6.0]	2.5 [1.5–3.5]	6.0 [5.5–6.5]	5.0 [3.5–6.0]	3.5 [2.0–4.5]	2.5 [2.0–3.5]	4.5 [3.5–6.0]

EDSS, Expanded Disability Status Scale; PP, primary progressive; RR, relapsing–remitting; SP, secondary progressive.

^a^Median and first and third interquartile.

The training and application cohorts significantly differed in baseline age, disease duration and EDSS (*P <* 0.0001). This result was expected because we split the cohort assigning the positive trials to the application cohort as we were interested in testing the treatment effect. Compared with the training cohort, at baseline, participants in the application cohort were significantly younger [mean 39.4 (9.5) versus mean 45.0 (10.5)], with shorter disease duration [mean 3.6 (4.5) years versus mean 8.7 (8.1) years], and more clinically impaired [median EDSS 4.5 (interquartile rage: 3.0–6.0) versus median EDSS 3.5 (2.0–4.5)], all *P* < 0.0001, and included more males (*P <* 0.005) (Table 1).

### The ICA-identified components were consistent between cohorts

**Figure 2:**
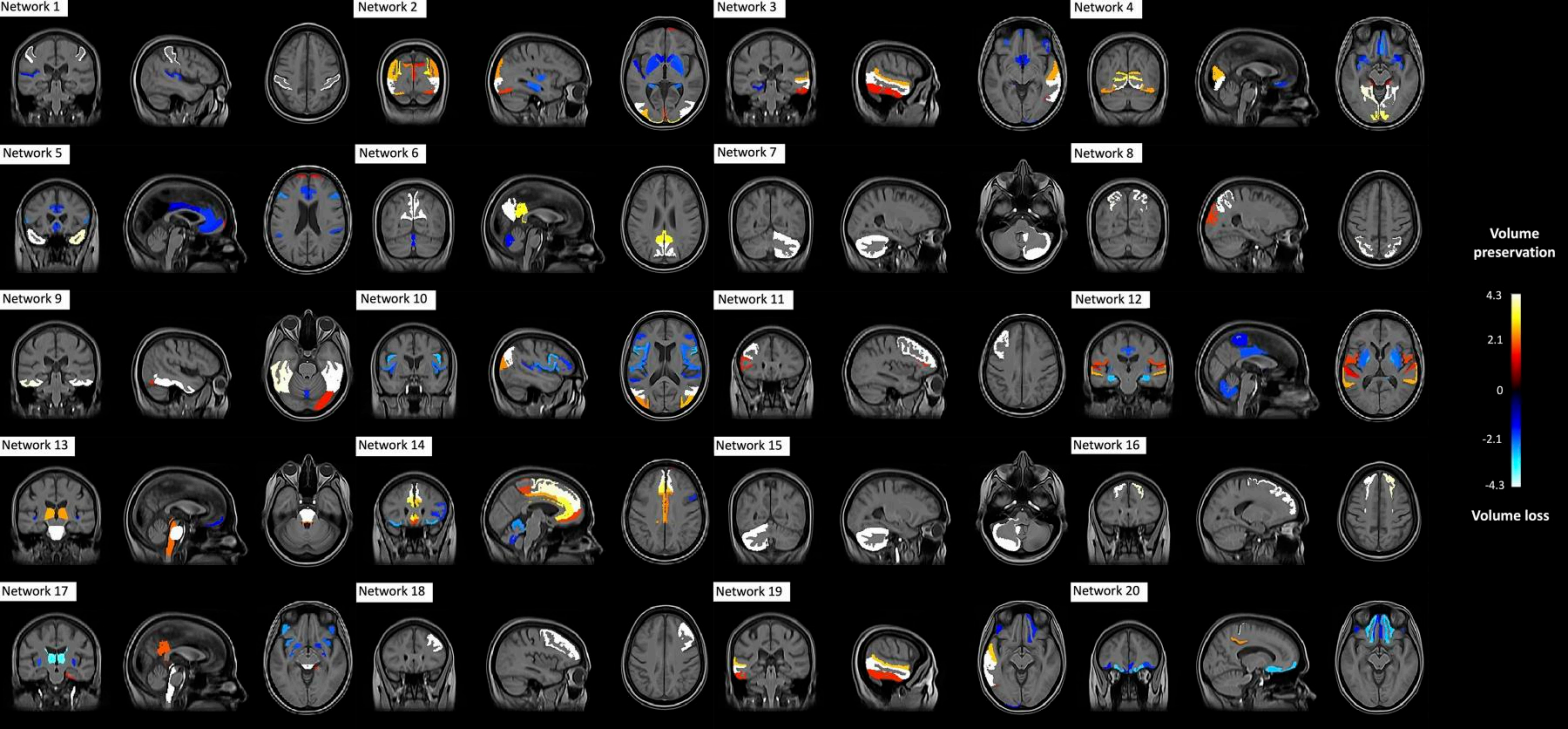
**Network of co-varying GM regional volumes.** We identified 20 networks of co-varying GM regions. Higher loadings represent higher GM content (brain volume preservation), while lower loadings indicate lower GM content (brain volume loss).

We identified 20 networks of co-varying GM regions (Fig. 2). Spatial cross-correlations between the ICA networks from the training and application cohorts ranged from 0.53 to 1.00 (see Supplementary Table 2). For 17 of these networks, the coefficient of correlation was ≥0.8, and for the remaining three, the coefficient of correlation ranged from 0.53 to 0.75. For a complete description of brain regions involved in each network, see Supplementary Table 3.

### The Lasso model replicated ICA loading factors at an individual level

Intra-class correlation coefficients for Lasso model were greater than 0.99 for all networks. We, therefore, present findings from the Lasso model outputs below, unless otherwise specified.

### Group comparison of GM network measures at baseline, and changes over time, across multiple sclerosis phenotypes

See Supplementary Table 4 for the coefficients and *P*-values for each network. In the training cohort at baseline, the Lasso model-derived loading factors in two networks (13 and 17) significantly differed between people with SP multiple sclerosis and PP multiple sclerosis, and between people with PP multiple sclerosis and RR multiple sclerosis, but not between people with RR multiple sclerosis and SP multiple sclerosis. The loading factor of Network 5 differed between people with RR multiple sclerosis and PP multiple sclerosis and between people with RR multiple sclerosis and SP multiple sclerosis, but not between people with SP multiple sclerosis and PP multiple sclerosis. In addition, the loading factor of four networks (7, 11, 12 and 15) differed between people with RR multiple sclerosis and people with SP multiple sclerosis. Whole-brain GM volumes also differed between RR multiple sclerosis and SP multiple sclerosis and between PP multiple sclerosis and RR multiple sclerosis.

**Figure 3:**
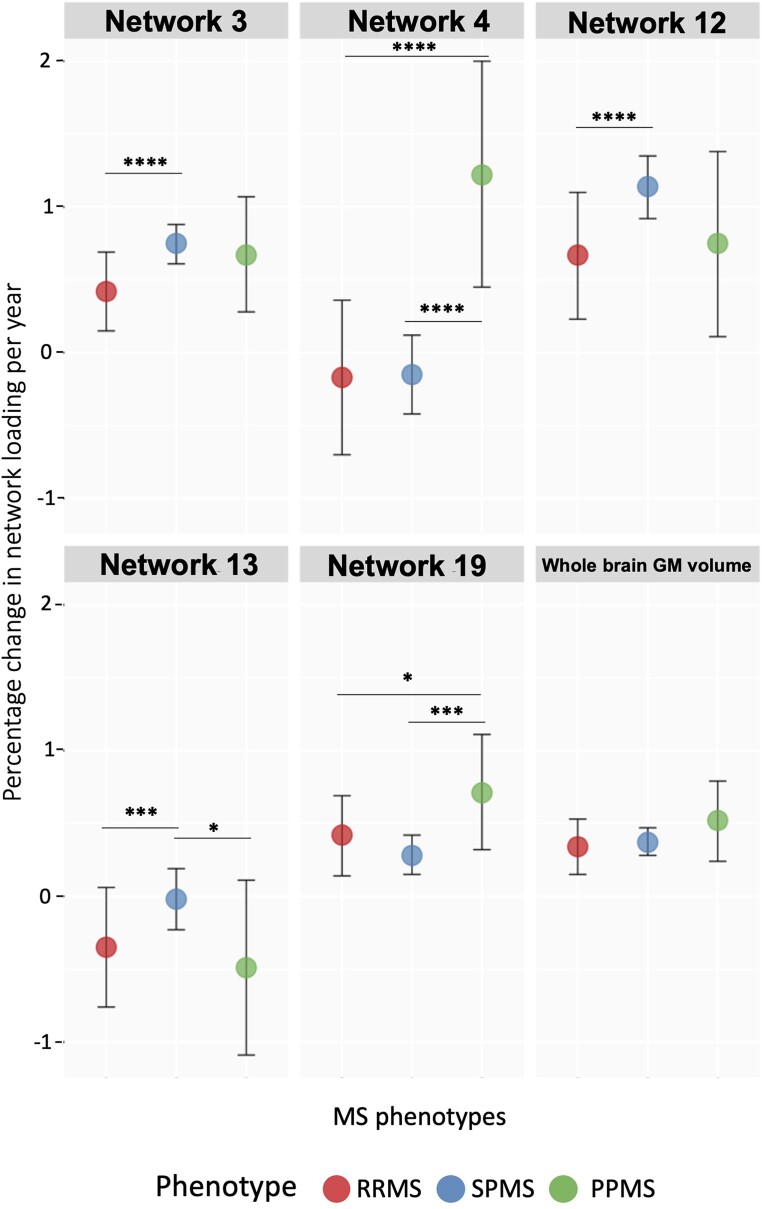
**Examples of the rate of GM volume loss across multiple sclerosis phenotypes over time (see Supplementary Fig. 1 for all networks**). We report here an example of the different rates of change in GM networks among multiple sclerosis phenotypes over time. To investigate differences in GM networks measures over time across multiple sclerosis phenotypes, in the training cohort, we used mixed-effect models with Lasso loading factors of each network as the dependent variable. The independent variables included multiple sclerosis phenotype, time (years from baseline) and the interaction between multiple sclerosis phenotype and time as fixed effects, baseline age, sex, disease duration, total intracranial volume and treatment as covariates. We used nested random effects models, in which visit was nested within each subject. Network 3 differed between participants with SP multiple sclerosis and RR multiple sclerosis, showing a greater annual percentage change in network loading (more GM atrophy) in people with SP multiple sclerosis than in RR multiple sclerosis (annual percentage change: 0.75% versus 0.42%, *β*: 0.003, CI [0.002:0.005], *P* < 0.0001). Networks 4 and 19 over time differed between people with PP multiple sclerosis and those with RR multiple sclerosis and SP multiple sclerosis, with a faster volume loss rate in people with PP multiple sclerosis [Network 4: annual percentage change of 1.22% versus −0.17% versus −0.15%, *β*: −0.014, CI (−0.018:0.009), *P* < 0.0001; *β*: −0.014, CI (−0.018:0.009), *P* < 0.0001; Network 19: annual percentage change of 0.72% versus 0.42% versus 28%, *β*: −0.004, CI (−0.007:−0.002), *P* < 0.005; *β*: −0.003, CI (−0.005:−0.001), *P* < 0.05]. Network 12 differed between participants with SP multiple sclerosis and RR multiple sclerosis, showing a greater annual percentage change in network loading in people with SP multiple sclerosis than in RR multiple sclerosis [Network 12: annual percentage change of 1.14% versus 0.67%, *β*: 0.005, CI (0.003:0.007), *P* < 0.005]. Network 13 differed between participants with SP multiple sclerosis and those with RR multiple sclerosis and PP multiple sclerosis, with a faster volume loss rate in people with SP multiple sclerosis [Network 13: annual percentage change of −0.02% versus −0.35% versus −0.49%, *β*: 0.003, CI (0.001:0.005), *P* < 0.005; *β*: 0.005, CI (0.001:0.009), *P* < 0.05). Whole-brain GM volume did not differ among multiple sclerosis phenotypes after correcting for multiple comparisons. ****P < 0.0001; ***P < 0.005; **P < 0.01; and *P < 0.05. *β*, beta coefficient; CI, confidence interval; GM, grey matter; MS, multiple sclerosis; RRMS, relapsing–remitting multiple sclerosis; SPMS, secondary progressive multiple sclerosis; PPMS, primary progressive multiple sclerosis.

Figure 3 shows an exemplar of how the loading factor rate changes across multiple sclerosis phenotypes. Over time, three of the identified networks showed greater volume loss in people with PP multiple sclerosis than those with SP multiple sclerosis and RR multiple sclerosis (Networks 4, 9 and 19), and two showed a greater volume loss in SP multiple sclerosis than in people with RR multiple sclerosis and PP multiple sclerosis (Networks 13 and 17). In two of these networks (3 and 12), volume loss was faster in SP multiple sclerosis than in RR multiple sclerosis, and the annual change in the loading factor of Network 8 was faster in SP multiple sclerosis and RR multiple sclerosis than in PP multiple sclerosis. Network 14 should have a greater volume loss in people with RR multiple sclerosis than SP multiple sclerosis, Network 7 a faster volume loss in people with SP multiple sclerosis than in PP multiple sclerosis and Network 17 in people with RR multiple sclerosis than in PP multiple sclerosis (Supplementary Table 4, Fig. 3, and Supplementary Fig. 1).

Substituting the Lasso-generated network measures for the original ICA loading factors yielded very similar results (Supplementary Table 5).

### GM network measures are associated with disability and clinical progression in multiple sclerosis

**Figure 4:**
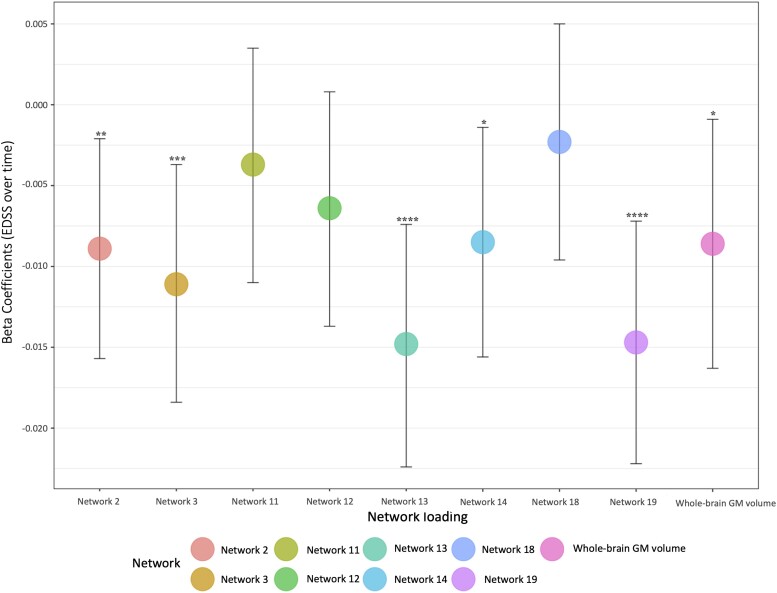
**Networks of co-varying GM regional volumes and whole-brain GM volume measures explain disability over time.** Figure shows associations between clinical disability (EDSS) and MRI measures. To determine whether network loading factors could explain disability progression, in the training cohort, we built mixed-effect models with EDSS as the dependent variable and GM network loading factor (as *z*-scores), time and the interaction between them; baseline age, sex, disease duration, total intracranial volume and treatment as independent, fixed-effect variables. Over time, five GM networks (2, 3, 13, 14 and 19) and the volume of the whole-brain GM were associated with the progression of disability (higher EDSS score) [Network 2: *β* = −0.01, 95% CI (−0.02:−0.002), *P* < 0.01; Network 3: *β* = −0.011, 95% CI (−0.02:−0.004), *P* < 0.005; Network 13: *β* = −0.02, 95% CI (−0.02:−0.01), *P* < 0.0001; Network 14: *β* = −0.009, 95% CI (−0.02:−0.001) *P* < 0.05; Network 19: *β* = −0.015, 95% CI (−0.02:−0.01), *P* < 0.0001; whole-brain GM: *β* = −0.01, 95% CI (−0.02:−0.01), *P* < 0.05]. ****P < 0.0001; ***P < 0.005; **P < 0.01; and *P < 0.05. *β*, beta coefficient; CI, confidence interval; EDSS, Expanded Disability Status Scale; GM, grey matter; MRI, magnetic resonance imaging.

Using the Lasso loading factors, in the training cohort at baseline, network measures were not associated with clinical disability (Table 2), while baseline EDSS scores were associated with whole-brain GM volume [*β* = −0.03, 95% confidence interval (CI) (−0.05:−0.01), *P <* 0.05] (Table 2 and Fig. 4).

**Table 2:** Cross-sectional and longitudinal association of clinical disability (EDSS) with GM network-based measures (obtained using Lasso), and whole-brain GM measures, in the training cohort

		Entire cohort (*β*, 95% CI, *P*)
Network 2	Baseline	−0.004, −0.029:0.022, *P* = 0.78
	Rate of change	−0.009, −0.016:−0.002, *P* < 0.01*
Network 3	Baseline	−0.030, −0.070:0.009, *P* = 0.14
	Rate of change	−0.011, −0.018:−0.004, *P* < 0.005*
Network 11	Baseline	−0.017, −0.058:0.023, *P* = 0.40
	Rate of change	−0.004, −0.011:0.004, *P* = 0.32
Network 12	Baseline	−0.036, −0.070:−0.002, *P* < 0.05
	Rate of change	−0.006, −0.014:0.001, *P* = 0.08
Network 13	Baseline	−0.024, −0.059:0.011, *P* = 0.19
	Rate of change	−0.015, −0.022:−0.007, *P* < 0.0001*
Network 14	Baseline	−0.026, −0.056:0.005, *P* = 0.10
	Rate of change	−0.009, −0.016:−0.001, *P* < 0.05*
Network 18	Baseline	−0.017, −0.059:0.025, *P* = 0.43
	Rate of change	−0.002, −0.010:0.005, *P* = 0.53
Network 19	Baseline	−0.045, −0.081:−0.009, *P* < 0.05
	Rate of change	−0.015, −0.022:−0.007, *P* < 0.0001*
GM volume	Baseline	−0.18, −0.25:−0.11, *P* < 0.0001*
	Rate of change	−0.009, −0.016:−0.001, *P* < 0.05*

*P-values that remained significant after correcting for multiple comparisons with the false discovery rate using Benjamini-Hochberg procedure.

When we looked at associations between GM network measures and EDSS over time, five networks (2, 3, 13, 14 and 19), and whole-brain GM volumes, were associated with EDSS changes over time [Network 2: *β* = −0.01, 95% CI (−0.02:−0.002), *P* < 0.01; Network 3: *β* = −0.011, 95% CI (−0.02:−0.004), *P* < 0.005; Network 13: *β* = −0.02, 95% CI (−0.02:−0.01), *P* < 0.0001; Network 14: *β* = −0.009, 95% CI (−0.02:−0.001), *P* < 0.05; Network 19: *β* = −0.015, 95% CI (−0.02:−0.01), *P* < 0.0001; whole-brain GM: *β* = −0.01, 95% CI (−0.02:−0.01), *P* < 0.05] (Table 2 and Fig. 4).

Results were similar when using the ICA network measures rather than Lasso loading factors (Supplementary Table 6).

In stepwise regression models, the final model explaining the disability progression, the final model explaining the disability progression included two networks (13 and 19), whole-brain GM volume, baseline age and disease duration (adjusted *R*^2^: 0.37). A regression model including only whole-brain GM volume, baseline age and disease duration had an adjusted *R*^2^ of 0.35.

When we repeated the analyses in the application cohort, both with Lasso network measures (trained using the ICA loading factors from the training cohort) and ICA loading factors (from the ICA in the application cohort), consistently the loading factor of Network 19, and whole-brain GM measures were associated with baseline disability. Disability progression was not associated with factor loadings or whole-brain GM volume measures (Supplementary Tables 7 and 8).

### GM network-based measures are sensitive to treatment effects

**Figure 5:**
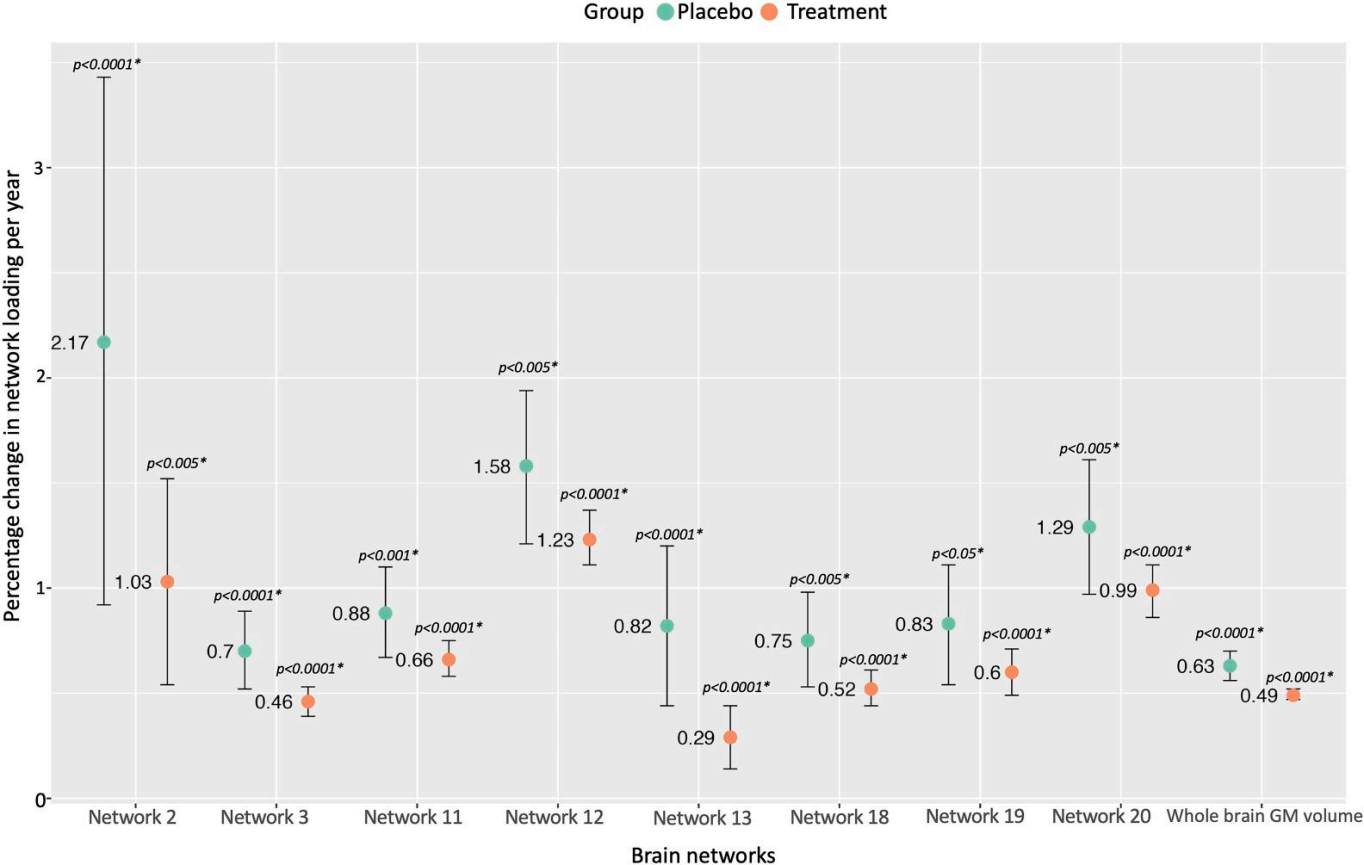
**Networks of co-varying GM and whole-brain GM volume measures show a treatment effect.** To determine whether network-based measures could detect treatment effects, we built a mixed-effect model with the interaction between time and treatment arm, baseline age, sex and total intracranial volume as fixed-effect variables and network measures as outcomes. Over time, there is a general decrease in GM volumes. However, eight of the identified GM networks, and whole-brain GM, showed a treatment effect: treated participants had relatively preserved GM (smaller annual percentage change) compared with those in the placebo arm. For instance, Network 13 showed a 0.29% annual percentage change in the treated group [*β*: −0.003, CI (−0.004:−0.001), *P* < 0.0001] compared with a 0.82% annual percentage change in the comparator arm [*β*: −0.005, CI (−0.008:−0.003), *P* < 0.0001]. ****P < 0.0001; ***P < 0.005; **P < 0.01; and *P < 0.05. *β*, beta coefficient; CI, confidence interval; GM, grey matter.

Using the Lasso network measures, in the application cohort, changes in eight GM networks, and whole-brain GM volumes, differed between the treated and the comparator groups (Fig. 5; Supplementary Table 9). Participants in the treated group, compared with comparator arm, had significantly lower annual percentage reductions in GM volumes in Network 2 (1.03% versus 2.17%), 3 (0.46% versus 0.70%), 11 (0.66% versus 0.88%), 12 (1.23% versus 1.58%), 13 (0.29% versus 0.82%), 18 (0.52% versus 0.75%), 19 (0.60% versus 0.83%) and 20 (0.99% versus 1.29%). The magnitude of treatment–placebo differences in the network-based measures was consistently greater for the GM networks compared with whole-brain GM volume measures (GM network treated versus placebo differences ranging from 1.14% to 0.22%, compared with 0.14% at a whole-brain level). Results were similar when using the loading factors derived from the ICA in this cohort (Supplementary Table 10). Sample size estimates, based on the observed differences between treated and comparator arm groups, over an average of 2.1 (0.9) years, with an alpha of 0.05 and power 80%, were lower in five of the eight Lasso-derived network-based measures (from 227 to 565 participants per arm) compared with whole-brain GM volume measures (753 participants per arm) (Supplementary Table 1^1^).

## Discussion

Using ICA, we identified multiple networks of co-varying GM volumes that differ between multiple sclerosis phenotypes at baseline and evolve more rapidly over time in PP multiple sclerosis and SP multiple sclerosis than in RR multiple sclerosis. Multiple network measures were associated with baseline clinical disability and disability progression. A Lasso model, based on the ICA network measures, when prospectively applied to unseen data from positive clinical trials was able to detect treatment effects that were of greater magnitude than those seen with whole-brain measures.

We found 20 networks of co-varying GM regional volumes, 17 of which were highly (*r* ≥ 0.8) consistent between the training and application cohorts. In the training cohort, at baseline, the loading factors in two networks differed between people with SP multiple sclerosis and PP multiple sclerosis and between people with PP multiple sclerosis and RR multiple sclerosis. Additionally, the loading factors of five networks differed between people with RR multiple sclerosis and those with progressive multiple sclerosis, while four networks differed between participants with RR multiple sclerosis and SP multiple sclerosis. Over time, people with SP multiple sclerosis showed greater volume loss than those with RR multiple sclerosis in four networks and those with PP multiple sclerosis in four networks. Participants with PP multiple sclerosis had greater volume loss than those with SP multiple sclerosis in three networks and those with RR multiple sclerosis in other three networks. People with RR multiple sclerosis had a faster volume rate in Network 14 than people with SP multiple sclerosis and in the loading factors of two networks than those with PP multiple sclerosis. If GM atrophy can be attributed to a single process affecting the whole brain, albeit progressing at different rates regionally, then we would have expected the same networks to differentiate multiple sclerosis phenotypes cross-sectionally and to change longitudinally, but this is not what we have observed. Instead, our results suggest that, while there are some common regional effects, there are some that are at least partly independent and phenotypically specific. It is possible that tract-mediated effects of white matter lesions could explain this, and it is already known that the distribution of white matter lesion differs between multiple sclerosis phenotypes.^36^ It would be of interest to see if a disconnectome analysis^37^ reveals spatially concordant cortical GM regions that also differ between multiple sclerosis phenotypes. However, it is also possible that multiple mechanisms are at work, including some regionally targeted neurodegenerative processes.^9,38,39^ Further work is now required to understand the pathological processes underlying these dynamic network-based differences and whether they represent multiple sclerosis phenotype-specific treatment targets.

We found that some, but not all, networks of GM volume loss could explain disability progression, as assessed by EDSS. When we consider that the EDSS is essentially a lower limb motor score in the range covered by the cohort included in this study (median EDSS 4.0, range 2.5–5.5), we would expect pathology affecting the motor network to be most relevant. Of the networks that correlated with disability, three out of five overlapped with motor network regions, spanning the cerebellum, thalamus, putamen, pallidum, supplementary motor cortex and supramarginal and postcentral gyri. This result suggests that pathology needs to occur in a clinically eloquent network to have detectable effects, and so not all atrophy will necessarily correlate with a given clinical outcome measure.

Eight GM network-based measures showed treatment effect with a lower percentage annual change in participants from the treated arm than those in the comparator group, and for five of these, the estimated cohort sizes were lower than for whole-brain GM volumes. This was most noticeable for Networks 11 and 13 (requiring cohorts of 477 and 227, respectively, compared with 753 for whole-brain GM volumes), which span brain regions involved in sensory and motor functions, suggesting that such targeted measures are potentially substantially more sensitive treatment effects in multiple sclerosis. The whole-brain measure used in this study was obtained using Geodesic Information Flows, making it directly comparable with the network-based measures, but in many clinical studies whereas SIENA is used, which assesses volume changes directly between images, rather than computing volumes and then subtracting, and this yields potentially more sensitive measures and, therefore, reduces the required sample size. However, using SIENA for this study would not be feasible (5089 participants over 22 045 visits). This is because pairwise comparisons between all visits for each patient are required, and for example, a patient with five visits would introduce more than 10 pairwise comparisons. Using volumetric information with mixed-effect models is more statistically efficient, which is why we chose to use it.

It would be of interest to combine direct measures of change in regional cortical volumes (this is not currently a function of SIENA) and a network-based approach, to see if this further increases potential sensitivity to treatment effects.

Recalling that ICA can only be run at a whole cohort level, i.e. it cannot be applied to unseen data, we developed a Lasso model to replicate the ICA network measures from the source GM regional volumes. The Lasso model achieved intra-class correlation scores of 0.99 with the ICA network measures, and the results (multiple sclerosis phenotype differences, associations with disability and treatment effects) using both Lasso and ICA network measures were materially the same. Further, the Lasso model (trained on a separate cohort) could be applied to another to look for treatment effects, and again results were materially the same as those derived from an ICA in the application cohort. While ideally an ICA would be undertaken when the whole cohort completes a study, the results suggest that the Lasso model measures are a reasonable substitute, and they have the advantage that they can be used in interim analyses or decision-making (for example in multi-arm clinical trials^40^).

This study was based on clinical trial data, using multiple treatment agents, studied over many years, MRI scanners and sites. This will have added variability to the imaging and clinical data that could have reduced sensitivity to regional atrophy and clinical associations, but equally it gives us confidence that the regional networks of GM atrophy we have identified are not artefacts of specific cohorts or MRI scanners and that it is realistic to expect that the method we have developed can be used in clinical trials. The training and application cohorts were deliberately different, as considered earlier. This meant that the Lasso model built using the ICA loading factors from the training cohort would not have been optimized for the application cohort, and this could have reduced sensitivity to clinical associations. In practice, this effect appears to have been small, given that we were able to replicate all the findings from the Lasso analyses with loading factors from an ICA run in the application cohort despite the demographic differences in cohorts. Moreover, we decided to use data from clinical trials that did not report a positive effect in the training cohort and those that showed a treatment effect in the validation cohort because we did not want results to be biased by the treatment effect. While mixing the trials would provide improved performance, there is a risk that this would come from over-fitting rather than from a true improvement.^41^ Moreover, mixing patients from the same trial across the train and test sets would introduce several issues: (i) it affects the underlying randomization process, and this would provide biased treatment effects or even misleading ones; (ii) data leakage: by separating the train and test, our approach ensures that our models not only generalize to new groups or cohorts (generalizability) but also have ‘transportability’ meaning that they can be applied in completely new settings. We applied ICA to regional GM volume measures, rather than running voxel- and surface-based analysis. A voxel or surface-based analysis may increase sensitivity to regional effects that do not match one of the Neuromorphometric Atlas regions; however, this approach also has drawbacks. For example, they require images to be registered to a common space (which can also reduce sensitivity to regional effects due to image smoothing) and they would have required a considerably higher computational power (which would currently be impractical for clinical studies). Because we included relatively more data from participants with progressive than RR multiple sclerosis, our cohort was older than the general multiple sclerosis population and network measures might also have been influenced by the effect of ageing. While we corrected all statistical analysis for the effect of age, future studies should investigate the age-specific dynamics in network measures and address network measures after stratifying the cohort according to age.

## Conclusion

In conclusion, the significant differences in GM network-based atrophy measures observed between multiple sclerosis phenotypes and their associations with clinical progression suggest that multiple pathological processes may underlie neurodegeneration in multiple sclerosis. Differences between the RR multiple sclerosis and SP multiple sclerosis groups suggest that SP multiple sclerosis is not simply a continuation of patterns of pathology seen in RR multiple sclerosis. Further work will be needed to determine the processes underlying these patterns and how they can best be targeted with treatments. Practically, this work shows that longitudinal network-based analyses of GM atrophy are feasible using clinical trials data and that they show treatment effects.

## Supplementary Material

fcae234_Supplementary_Data

## Data Availability

Data used in this work are controlled by several pharmaceutical companies and cannot be shared directly. Applications for access should be directed to the companies controlling each dataset. The computer codes to obtain longitudinal individual-level network-based measures can be found at: https://github.com/co-el/Longitudinal_individual_level_structural_network.
