# Supplementary material for: Longitudinal network-based brain grey matter MRI measures are clinically relevant and sensitive to treatment effects in multiple sclerosis
Source: Brain Commun. 2024 Jul 29;6(4):fcae234. doi: 10.1093/braincomms/fcae234 (PMC11285187; doi:10.1093/braincomms/fcae234)
Supplement: fcae234_Supplementary_Data [file fcae234_supplementary_data.pdf]

Supplementary Materials

Appendix 1

International Progressive MS Alliance (IPMSA) Investigators in this network:

Name	Institution
Douglas L. Arnold	McConnell Brain Imaging Centre, Montreal Neurological Institute, McGill University, Montreal, Quebec, Canada
Sridar Narayanan	McConnell Brain Imaging Centre, Montreal Neurological Institute, McGill University, Montreal, Quebec, Canada
Frederik Barkhof	Queen Square Multiple Sclerosis Centre, Department of Neuroinflammation, UCL Queen Square Institute of Neurology, Faculty of Brain Sciences, University College London, WC1B5EH, UK
Olga Ciccarelli	Queen Square Multiple Sclerosis Centre, Department of Neuroinflammation, UCL Queen Square Institute of Neurology, Faculty of Brain Sciences, University College London, WC1B5EH, UK
Declan Chard	Queen Square Multiple Sclerosis Centre, Department of Neuroinflammation, UCL Queen Square Institute of Neurology, Faculty of Brain Sciences, University College London, WC1B5EH, UK
Louis Collins	McConnell Brain Imaging Centre, Montreal Neurological Institute, McGill University, Montreal, Quebec, Canada
Tal Arbel	McConnell Brain Imaging Centre, Montreal Neurological Institute, McGill University, Montreal, Quebec, Canada
Charles R.G. Guttman	Center for Neurological Imaging, Brigham and Women’s Hospital, Harvard Medical School, Massachusetts, USA
Jerry S. Wolinsky	McGovern Medical School, The University of Texas Health Science Center at Houston (UTHealth), Houston, Texas, USA
Garry R. Cutter	University of Alabama at Birmingham School of Public Health, USA
Nicola De Stefano	University of Siena, Italy
Maria Pia Sormani	University of Genoa, Italy
Ludwig Kappos	University Hospital Basel, Switzerland
Jack H Simon	Oregon Health and Sciences University, Portland Veterans Affairs Medical Center, Oregon, USA
Jeremy Chataway	Queen Square Multiple Sclerosis Centre, Department of Neuroinflammation, UCL Queen Square Institute of Neurology,

	Faculty of Brain Sciences, University College London, WC1B5EH, UK
Raj Kapoor	Queen Square Multiple Sclerosis Centre, Department of Neuroinflammation, UCL Queen Square Institute of Neurology, Faculty of Brain Sciences, University College London, WC1B5EH, UK
Howard L. Weiner (CLIMB Investigator)	Brigham and Women's Hospital, Ann Romney Center for Neurologic Diseases, Department of Neurology, Boston, MA, 02115
Tanuja Chitnis (CLIMB Investigator)	Brigham and Women's Hospital, Ann Romney Center for Neurologic Diseases, Department of Neurology, Boston, MA, 02115
Rohit Bakshi (CLIMB Investigator)	Brigham and Women's Hospital, Ann Romney Center for Neurologic Diseases, Department of Neurology, Boston, MA, 02115

Supplementary Table 1 Details of clinical trials included in this study

Trial	ClinicalTrials.gov Identifier	Sample	MS type	Age Mean (SD)	Sex (M/F)	EDSS Median (range)	Trial drug	Duration of follow-up (years) mean(SD)	End-point achieved	Cohort
**ASCEND**	NCT00135226	851	SPMS	46.7 (7.7)	319/532	6.0 [3.0-6.5]	Natalizumab 300 mg	12.0 (6.9)	No	Training
**DCE**	NCT00420212	1060	RRMS	37.5 (9.1)	293/767	2.5 [0.0-6.0]	Glatiramer Acetate, BG00012 240 mg TID, BG00012 240 mg BID	4.9 (5.2)	No	Training
**LIPOIC-ACID**	NCT03161028	37	SPMS	58.8 (6.2)	14/23	6.0 [3.0-9.0]	Lipoic-acid	29.3 (9.3)	No	Training
**MS-SMART**	NCT01910259	394	SPMS	54.7 (7.0)	131/263	6.0 [4.0-6.5]	Fluoxetine, Riluzole, Amiloride	15.2 (9.4)	No	Training
**OLYMPUS**	NCT00087529	422	PPMS	49.9 (8.9)	209/213	5.0 [2.0-7.0]	Rituxumab	4.0 (4.3)	No	Training
**OPERA1**	NCT01412333	800	RRMS	37.0 (9.4)	271/529	2.5 [0.0-6.0]	Interferon B-1a, Ocrelizumab	3.9 (4.8)	Yes	Application
**OPERA2**	NCT01412333	824	RRMS	37.3 (9.0)	283/541	2.5 [0.0-6.0]	Interferon B-1a, Ocrelizumab	4.2 (5.0)	Yes	Application
**ORATORIO**	NCT01194570	701	PPMS	44.6 (8.0)	353/348	4.5 [2.5-6.5]	Ocrelizumab	2.83 (3.1)	Yes	Application

Supplementary Table 2 Spatial cross-correlations between GM components identified from the training and application cohorts.

Networks	Spatial cross-correlations
1	r= 0.90
2	r= 0.80
3	r= 0.92
4	r= 0.84
5	r= 0.82
6	r= 0.92
7	r= 1.00
8	r= 0.90
9	r= 0.92
10	r= 0.94
11	r= 0.97
12	r= 0.75
13	r= 0.72
14	r= 0.91
15	r= 1.00
16	r=1.00
17	r= 0.53
18	r= 0.94
19	r= 0.95
20	r= 0.96

Supplementary Table 3 Brain regions involved in each GM network.

Network	Brain regions
Network 1	Transverse temporal gyrus, planum temporale, postcentral gyrus
Network 2	Cerebellum, inferior, middle, and superior occipital gyrus, fusiform gyrus, cuneus, frontal pole, accumbens area, subcallosal area, planum polare, cingulate gyrus, caudate, pallidum, putamen, parahippocampal gyrus, amygdala, hippocampus
Network 3	Postcentral gyrus, left anterior orbital gyrus, right occipital pole, left frontal pole, left gyrus rectus, right lateral orbital gyrus, accumbens area, basal forebrain, subcallosal area, occipital fusiform gyrus, parahippocampal gyrus, cerebellum, amygdala, inferior, middle, and superior temporal gyrus.
Network 4	Medial frontal cortex, orbital, opercular, and triangular part of the inferior frontal gyrus, frontal operculum, insula, gyrus rectus, transverse temporal gyrus, claustrum, medial frontal cortex, parietal operculum, planum temporale, central and parietal operculum, planum temporale, frontal pole, parahippocampal gyrus, occipital fusiform gyrus, cuneus, calcarine cortex, lingual gyrus
Network 5	Triangular part of the inferior frontal gyrus, parietal operculum, anterior and middle cingulate gyrus, medial frontal cortex, transverse temporal gyrus, frontal pole, enthorhinal area, temporal pole
Network 6	Precuneus, posterior cingulate gyrus, anterior and lateral orbital gyrus, pallidum, cerebellum
Network 7	Right cerebellum
Network 8	Planum polare, superior temporal gyrus, superior parietal lobule, superior occipital gyrus, anterior insula
Network 9	Inferior temporal gyrus, fusiform gyrus, medial orbital gyrus, occipital fusiform gyrus, cerebellum, pallidum, calcarine cortex, postcentral gyrus, forebrain, accumbens area, postcentral gyrus
Network 10	Angular gyrus, middle occipital gyrus, planum temporale, transverse temporal gyrus, triangular and opercular part of the inferior frontal gyrus, anterior insula, frontal and central operculum
Network 11	Planum polare, posterior insula, triangular part of the inferior and middle frontal gyrus, postcentral gyrus
Network 12	Hippocampus, pallidum, amygdala, putamen, claustrum, subcallosal area, middle cingulate gyrus, cerebellum, entorhinal area, precentral gyrus, posterior orbital gyrus, basal forebrain, accumbens, planum temporale, central operculum, superior temporal gyrus, supramarginal gyrus
Network 13	Pons, brain stem, thalamus, planum polare, putamen, orbital part of the inferior frontal gyrus, gyrus rectus, anterior orbital gyrus
Network 14	Posterior orbital gyrus, cerebellum, planum temporale and polare, triangular and opercular part of the inferior and superior frontal gyrus, transverse temporal gyrus, parietal operculum, frontal pole, medial frontal cortex, precentral gyrus, anterior and middle cingulate gyrus, supplementary motor cortex
Network 15	Left cerebellum
Network 16	Right superior frontal gyrus

Network 17	Caudate, thalamus, lateral orbital gyrus, frontal pole, anterior insula, putamen, parahippocampal gyrus, posterior cingulate gyrus, entorhinal area, brain stem
Network 18	Right middle frontal gyrus
Network 19	Inferior, middle, and superior temporal gyrus, lateral and medial orbital gyrus, occipital pole
Network 20	Precentral gyrus, lateral and medial orbital gyrus, gyrus rectus, medial frontal cortex

Supplementary Table 4 Group comparison of GM networks among MS phenotypes and overtime in the training cohort using network-based measures obtained using Lasso.

**Network 1**			
**Group comparison**	**Cross-sectional**		
	β	*CI*	*p*
**SPMS vs. RRMS**	-0.006	-0.021:0.009	0.42
**SPMS vs. PPMS**	-0.005	-0.021:0.011	0.53
**RRMS vs. PPMS**	0.001	-0.016:0.018	0.91
**Group comparison**	**Longitudinal**		
	β	*CI*	*p*
**Rate of change in SPMS vs. RRMS**	0.0001	-0.001:0.001	0.99
**Rate of change in SPMS vs. PPMS**	0.0001	-0.003:0.003	0.82
**Rate of change in RRMS vs. PPMS**	0.0001	-0.002:0.003	0.81
**Network 2**			
**Group comparison**	**Cross-sectional**		
	β	*CI*	*p*
**SPMS vs. RRMS**	-0.013	-0.054:0.028	0.53
**SPMS vs. PPMS**	-0.021	-0.065:0.023	0.35
**RRMS vs. PPMS**	-0.008	-0.056:0.04	0.75
**Group comparison**	**Longitudinal**		
	β	*CI*	*p*
**Rate of change in SPMS vs. RRMS**	0.006	0.001:0.011	<0.05
**Rate of change in SPMS vs. PPMS**	-0.002	-0.012:0.007	0.63
**Rate of change in RRMS vs. PPMS**	-0.008	-0.017:0.001	0.07
**Network 3**			
**Group comparison**	**Cross-sectional**		
	β	*CI*	*p*
**SPMS vs. RRMS**	-0.006	-0.016:0.004	0.24
**SPMS vs. PPMS**	-0.005	-0.016:0.006	0.38
**RRMS vs. PPMS**	0.001	-0.011:0.013	0.84

Group comparison	Longitudinal		
	β	*CI*	*p*
**Rate of change in SPMS vs. RRMS**	0.003	0.002:0.005	<0.0001*
**Rate of change in SPMS vs. PPMS**	0.001	-0.002:0.003	0.58
**Rate of change in RRMS vs. PPMS**	-0.003	-0.005:0	<0.05
**Network 4**			
**Group comparison**	**Cross-sectional**		
	β	*CI*	*p*
**SPMS vs. RRMS**	0.002	-0.023:0.027	0.87
**SPMS vs. PPMS**	0.01	-0.017:0.037	0.46
**RRMS vs. PPMS**	0.008	-0.021:0.037	0.59
**Group comparison**	**Longitudinal**		
	β	*CI*	*p*
**Rate of change in SPMS vs. RRMS**	0.0001	-0.002:0.003	0.89
**Rate of change in SPMS vs. PPMS**	-0.014	-0.019:-0.009	<0.0001*
**Rate of change in RRMS vs. PPMS**	-0.014	-0.019:-0.009	<0.0001*
**Network 5**			
**Group comparison**	**Cross-sectional**		
	β	*CI*	*p*
**SPMS vs. RRMS**	0.075	0.02:0.13	<0.01*
**SPMS vs. PPMS**	-0.032	-0.091:0.027	0.29
**RRMS vs. PPMS**	-0.107	-0.172:-0.042	<0.005*
**Group comparison**	**Longitudinal**		
	β	*CI*	*p*
**Rate of change in SPMS vs. RRMS**	0.0001	-0.009:0.009	0.054
**Rate of change in SPMS vs. PPMS**	-0.005	-0.013:0.004	0.96
**Rate of change in RRMS vs. PPMS**	-0.004	-0.012:0.004	0.29
**Network 6**			
**Group comparison**	**Cross-sectional**		
	β	*CI*	*p*

**SPMS vs. RRMS**	-0.009	-0.026:0.009	0.33
**SPMS vs. PPMS**	-0.013	-0.032:0.006	0.18
**RRMS vs. PPMS**	-0.004	-0.025:0.016	0.70
**Group comparison**	**Longitudinal**		
	β	*CI*	*p*
**Rate of change in SPMS vs. RRMS**	-0.002	-0.003:0.001	<0.05
**Rate of change in SPMS vs. PPMS**	-0.003	-0.006:0.001	<0.05
**Rate of change in RRMS vs. PPMS**	-0.001	-0.004:0.001	0.32
**Network 7**			
**Group comparison**	**Cross-sectional**		
	β	*CI*	*p*
**SPMS vs. RRMS**	-0.02	-0.031:-0.008	<0.005*
**SPMS vs. PPMS**	-0.011	-0.024:0.002	0.10
**RRMS vs. PPMS**	0.009	-0.005:0.023	0.22
**Group comparison**	**Longitudinal**		
	β	*CI*	*P*
**Rate of change in SPMS vs. RRMS**	0.002	0:0.004	<0.05
**Rate of change in SPMS vs. PPMS**	0.004	0.001:0.008	<0.01*
**Rate of change in RRMS vs. PPMS**	0.003	-0.001:0.006	0.10
**Network 8**			
**Group comparison**	**Cross-sectional**		
	β	*CI*	*p*
**SPMS vs. RRMS**	0.009	-0.016:0.033	0.48
**SPMS vs. PPMS**	-0.021	-0.047:0.005	0.12
**RRMS vs. PPMS**	-0.029	-0.058:-0.001	<0.05
**Group comparison**	**Longitudinal**		
	β	*CI*	*p*
**Rate of change in SPMS vs. RRMS**	-0.002	-0.004:0	0.12
**Rate of change in SPMS vs. PPMS**	0.008	0.003:0.013	<0.001*
**Rate of change in RRMS vs. PPMS**	0.01	0.006:0.014	<0.0001*

Network 9			
**Group comparison**	**Cross-sectional**		
	β	*CI*	*p*
**SPMS vs. RRMS**	0.005	-0.004:0.014	0.25
**SPMS vs. PPMS**	0.002	-0.007:0.012	0.65
**RRMS vs. PPMS**	-0.003	-0.013:0.007	0.58
**Group comparison**	**Longitudinal**		
	β	*CI*	*p*
**Rate of change in SPMS vs. RRMS**	0.001	-0.001:0.001	0.89
**Rate of change in SPMS vs. PPMS**	-0.003	-0.006:-0.001	<0.01*
**Rate of change in RRMS vs. PPMS**	-0.003	-0.006:-0.001	<0.005*
**Network 10**			
**Group comparison**	**Cross-sectional**		
	β	*CI*	*p*
**SPMS vs. RRMS**	0.003	-0.019:0.026	0.77
**SPMS vs. PPMS**	0.001	-0.024:0.025	0.96
**RRMS vs. PPMS**	-0.003	-0.029:0.024	0.84
**Group comparison**	**Longitudinal**		
	β	*CI*	*p*
**Rate of change in SPMS vs. RRMS**	-0.002	-0.005:0	<0.05
**Rate of change in SPMS vs. PPMS**	-0.002	-0.006:0.003	0.43
**Rate of change in RRMS vs. PPMS**	0.001	-0.003:0.005	0.76
**Network 11**			
**Group comparison**	**Cross-sectional**		
	β	*CI*	*p*
**SPMS vs. RRMS**	-0.018	-0.03:-0.006	<0.005*
**SPMS vs. PPMS**	-0.001	-0.015:0.012	0.85
**RRMS vs. PPMS**	0.017	0.002:0.031	<0.05
**Group comparison**	**Longitudinal**		
	β	*CI*	*p*
**Rate of change in SPMS vs. RRMS**	0.001	-0.001:0.002	0.39

**Rate of change in SPMS vs. PPMS**	-0.002	-0.005:0.001	0.26
**Rate of change in RRMS vs. PPMS**	-0.002	-0.005:0	0.08
**Network 12**			
**Group comparison**	**Cross-sectional**		
	β	*CI*	*p*
**SPMS vs. RRMS**	-0.032	-0.051:-0.013	<0.005*
**SPMS vs. PPMS**	-0.004	-0.025:0.016	0.69
**RRMS vs. PPMS**	0.027	0.005:0.05	<0.05
**Group comparison**	**Longitudinal**		
	β	*CI*	*p*
**Rate of change in SPMS vs. RRMS**	0.005	0.003:0.007	<0.0001*
**Rate of change in SPMS vs. PPMS**	0.004	0:0.008	0.065
**Rate of change in RRMS vs. PPMS**	-0.001	-0.005:0.003	0.67
**Network 13**			
**Group comparison**	**Cross-sectional**		
	β	*CI*	*p*
**SPMS vs. RRMS**	-0.007	-0.027:0.013	0.48
**SPMS vs. PPMS**	-0.042	-0.064:-0.021	<0.0005*
**RRMS vs. PPMS**	-0.035	-0.059:-0.012	<0.005*
**Group comparison**	**Longitudinal**		
	β	*CI*	*p*
**Rate of change in SPMS vs. RRMS**	0.003	0.001:0.005	<0.005*
**Rate of change in SPMS vs. PPMS**	0.005	0.001:0.009	<0.05*
**Rate of change in RRMS vs. PPMS**	0.001	-0.002:0.005	0.43
**Network 14**			
**Group comparison**	**Cross-sectional**		
	β	*CI*	*p*
**SPMS vs. RRMS**	0.002	-0.027:0.031	0.90
**SPMS vs. PPMS**	0.002	-0.029:0.034	0.90

**RRMS vs. PPMS**	0.0001	-0.034:0.035	0.99
**Group comparison**	**Longitudinal**		
	β	*CI*	*p*
**Rate of change in SPMS vs. RRMS**	-0.005	-0.008:-0.002	<0.005*
**Rate of change in SPMS vs. PPMS**	-0.007	-0.013:0	<0.05
**Rate of change in RRMS vs. PPMS**	-0.001	-0.007:0.004	0.62
**Network 15**			
**Group comparison**	**Cross-sectional**		
	β	*CI*	*p*
**SPMS vs. RRMS**	-0.02	-0.032:-0.008	<0.001*
**SPMS vs. PPMS**	-0.01	-0.023:0.003	0.12
**RRMS vs. PPMS**	0.01	-0.004:0.024	0.16
**Group comparison**	**Longitudinal**		
	β	*CI*	*p*
**Rate of change in SPMS vs. RRMS**	0.001	0:0.003	0.07
**Rate of change in SPMS vs. PPMS**	0.005	0.003:0.008	<0.0005*
**Rate of change in RRMS vs. PPMS**	0.004	0.001:0.006	<0.005*
**Network 16**			
**Group comparison**	**Cross-sectional**		
	β	*CI*	*p*
**SPMS vs. RRMS**	-0.006	-0.018:0.007	0.38
**SPMS vs. PPMS**	-0.004	-0.017:0.01	0.59
**RRMS vs. PPMS**	0.002	-0.013:0.017	0.80
**Group comparison**	**Longitudinal**		
	β	*CI*	*p*
**Rate of change in SPMS vs. RRMS**	0.0001	-0.001:0.002	0.80
**Rate of change in SPMS vs. PPMS**	0.001	-0.002:0.004	0.48
**Rate of change in RRMS vs. PPMS**	0.001	-0.002:0.004	0.53
**Network 17**			

Group comparison	Cross-sectional		
	β	*CI*	*p*
**SPMS vs. RRMS**	-0.01	-0.031:0.012	0.38
**SPMS vs. PPMS**	-0.055	-0.078:-0.032	<0.0001*
**RRMS vs. PPMS**	-0.046	-0.071:-0.021	<0.0005*
**Group comparison**	**Longitudinal**		
	β	*CI*	*p*
**Rate of change in SPMS vs. RRMS**	0.008	0.005:0.011	<0.0001*
**Rate of change in SPMS vs. PPMS**	0.034	0.028:0.04	<0.0001*
**Rate of change in RRMS vs. PPMS**	0.025	0.02:0.031	<0.0001*
**Network 18**			
**Group comparison**	**Cross-sectional**		
	β	*CI*	*p*
**SPMS vs. RRMS**	-0.012	-0.025:0.001	0.07
**SPMS vs. PPMS**	-0.001	-0.015:0.013	0.89
**RRMS vs. PPMS**	0.011	-0.004:0.026	0.16
**Group comparison**	**Longitudinal**		
	β	*CI*	*p*
**Rate of change in SPMS vs. RRMS**	-0.001	-0.002:0.001	0.24
**Rate of change in SPMS vs. PPMS**	0.0001	-0.003:0.003	0.96
**Rate of change in RRMS vs. PPMS**	0.001	-0.002:0.003	0.54
**Network 19**			
**Group comparison**	**Cross-sectional**		
	β	*CI*	*p*
**SPMS vs. RRMS**	-0.005	-0.017:0.00	0.49
**SPMS vs. PPMS**	-0.002	-0.016:0.012	0.75
**RRMS vs. PPMS**	0.002	-0.013:0.017	0.77
**Group comparison**	**Longitudinal**		
	β	*CI*	*p*
**Rate of change in SPMS vs. RRMS**	-0.001	-0.003:0	0.052

**Rate of change in SPMS vs. PPMS**	-0.004	-0.007:-0.002	<0.005*
**Rate of change in RRMS vs. PPMS**	-0.003	-0.005:-0.001	<0.05*
**Network 20**			
**Group comparison**	**Cross-sectional**		
	β	*CI*	*p*
**SPMS vs. RRMS**	-0.012	-0.028:0.004	0.16
**SPMS vs. PPMS**	-0.018	-0.036:-0.001	<0.05
**RRMS vs. PPMS**	-0.007	-0.026:0.012	0.48
**Group comparison**	**Longitudinal**		
	β	*CI*	*p*
**Rate of change in SPMS vs. RRMS**	0.001	-0.001:0.002	0.49
**Rate of change in SPMS vs. PPMS**	0.0001	-0.003:0.004	0.80
**Rate of change in RRMS vs. PPMS**	0.0001	-0.003:0.003	0.90
**Whole brain grey matter volume**			
**Group comparison**	**Cross-sectional**		
	β	*CI*	*p*
**SPMS vs. RRMS**	-0.012	-0.017:-0.006	<0.0001*
**SPMS vs. PPMS**	-0.003	-0.009:0.002	0.23
**RRMS vs. PPMS**	0.008	0.002:0.014	<0.01*
**Group comparison**	**Longitudinal**		
	β	*CI*	*p*
**Rate of change in SPMS vs. RRMS**	0.0001	-0.001:0.001	0.52
**Rate of change in SPMS vs. PPMS**	-0.001	-0.003:0	0.13
**Rate of change in RRMS vs. PPMS**	-0.002	-0.003:0	<0.05

*Statistically significant after False Discovery Rate correction

Abbreviations: *β* = Beta Estimates; SPMS = Secondary Progressive Multiple Sclerosis; PPMS = Primary Progressive Multiple Sclerosis; RRMS = Relapsing Remitting Multiple Sclerosis; SE = Standard Error

Supplementary Table 5 Group comparison of GM networks among MS phenotypes and overtime in the training cohort using network-based measures obtained using ICA.

**Network 1**			
**Group comparison**	**Cross-sectional**		
	β	*CI*	*p*
**SPMS vs. RRMS**	-0.006	-0.021:0.009	0.42
**SPMS vs. PPMS**	-0.005	-0.021:0.011	0.53
**RRMS vs. PPMS**	0.001	-0.016:0.018	0.91
**Group comparison**	**Longitudinal**		
	β	*CI*	*p*
**Rate of change in SPMS vs. RRMS**	0.001	-0.001:0.001	0.99
**Rate of change in SPMS vs. PPMS**	0.001	-0.003:0.003	0.83
**Rate of change in RRMS vs. PPMS**	0.0001	-0.002:0.003	0.82
**Network 2**			
**Group comparison**	**Cross-sectional**		
	β	*CI*	*p*
**SPMS vs. RRMS**	-0.014	-0.054:0.027	0.52
**SPMS vs. PPMS**	-0.021	-0.065:0.022	0.34
**RRMS vs. PPMS**	-0.008	-0.056:0.04	0.75
**Group comparison**	**Longitudinal**		
	β	*CI*	*p*
**Rate of change in SPMS vs. RRMS**	0.006	0.001:0.011	<0.05
**Rate of change in SPMS vs. PPMS**	-0.002	-0.012:0.008	0.70
**Rate of change in RRMS vs. PPMS**	-0.008	-0.017:0.001	0.08
**Network 3**			
**Group comparison**	**Cross-sectional**		
	β	*CI*	*p*
**SPMS vs. RRMS**	-0.006	-0.016:0.004	0.23
**SPMS vs. PPMS**	-0.005	-0.016:0.006	0.38
**RRMS vs. PPMS**	0.001	-0.01:0.013	0.83

Group comparison	Longitudinal		
	β	*CI*	*p*
**Rate of change in SPMS vs. RRMS**	0.003	0.002:0.005	<0.0001*
**Rate of change in SPMS vs. PPMS**	0.001	-0.002:0.003	0.58
**Rate of change in RRMS vs. PPMS**	-0.003	-0.005:0	<0.05
**Network 4**			
**Group comparison**	**Cross-sectional**		
	β	*CI*	*p*
**SPMS vs. RRMS**	0.002	-0.023:0.027	0.87
**SPMS vs. PPMS**	0.01	-0.017:0.037	0.46
**RRMS vs. PPMS**	0.008	-0.021:0.037	0.59
**Group comparison**	**Longitudinal**		
	β	*CI*	*p*
**Rate of change in SPMS vs. RRMS**	0.0001	-0.002:0.003	0.90
**Rate of change in SPMS vs. PPMS**	-0.014	-0.019:-0.009	<0.0001*
**Rate of change in RRMS vs. PPMS**	-0.014	-0.019:-0.009	<0.0001*
**Network 5**			
**Group comparison**	**Cross-sectional**		
	β	*CI*	*p*
**SPMS vs. RRMS**	0.075	0.02:0.13	<0.005*
**SPMS vs. PPMS**	-0.031	-0.091:0.028	0.30
**RRMS vs. PPMS**	-0.107	-0.171:-0.042	<0.005*
**Group comparison**	**Longitudinal**		
	β	*CI*	*p*
**Rate of change in SPMS vs. RRMS**	0.005	0:0.009	<0.053
**Rate of change in SPMS vs. PPMS**	-0.001	-0.01:0.008	0.87
**Rate of change in RRMS vs. PPMS**	-0.005	-0.014:0.003	0.20
**Network 6**			
**Group comparison**	**Cross-sectional**		
	β	*CI*	*p*

**SPMS vs. RRMS**	-0.009	-0.026:0.009	0.32
**SPMS vs. PPMS**	-0.013	-0.032:0.006	0.18
**RRMS vs. PPMS**	-0.004	-0.025:0.016	0.32
**Group comparison**	**Longitudinal**		
	β	*CI*	*p*
**Rate of change in SPMS vs. RRMS**	-0.002	-0.003:0	<0.05
**Rate of change in SPMS vs. PPMS**	-0.003	-0.006:0	<0.05
**Rate of change in RRMS vs. PPMS**	-0.001	-0.004:0.001	0.33
**Network 7**			
**Group comparison**	**Cross-sectional**		
	β	*CI*	*p*
**SPMS vs. RRMS**	-0.02	-0.031:-0.008	<0.005*
**SPMS vs. PPMS**	-0.011	-0.024:0.002	0.10
**RRMS vs. PPMS**	0.009	-0.005:0.023	0.22
**Group comparison**	**Longitudinal**		
	β	*CI*	*p*
**Rate of change in SPMS vs. RRMS**	0.002	0:0.004	<0.05
**Rate of change in SPMS vs. PPMS**	0.005	0.001:0.008	<0.01*
**Rate of change in RRMS vs. PPMS**	0.003	0:0.006	0.09
**Network 8**			
**Group comparison**	**Cross-sectiona**		
	β	*CI*	*p*
**SPMS vs. RRMS**	0.009	-0.016:0.033	0.48
**SPMS vs. PPMS**	-0.021	-0.047:0.005	0.12
**RRMS vs. PPMS**	-0.029	-0.058:-0.001	<0.05
**Group comparison**	**Longitudinal**		
	β	*CI*	*p*
**Rate of change in SPMS vs. RRMS**	-0.002	-0.004:0	0.12
**Rate of change in SPMS vs. PPMS**	0.008	0.003:0.013	<0.0001*
**Rate of change in RRMS vs. PPMS**	0.01	0.006:0.014	<0.0001*

**Network 9**			
**Group comparison**	**Cross-sectional**		
	β	*CI*	*p*
**SPMS vs. RRMS**	0.005	-0.004:0.014	0.26
**SPMS vs. PPMS**	0.002	-0.007:0.012	0.65
**RRMS vs. PPMS**	-0.003	-0.013:0.007	0.59
**Group comparison**	**Longitudinal**		
	β	*CI*	*p*
**Rate of change in SPMS vs. RRMS**	0.0001	-0.001:0.001	0.90
**Rate of change in SPMS vs. PPMS**	-0.003	-0.006:-0.001	<0.05*
**Rate of change in RRMS vs. PPMS**	-0.003	-0.006:-0.001	<0.01*
**Network 10**			
**Group comparison**	**Cross-sectional**		
	β	*CI*	*p*
**SPMS vs. RRMS**	0.003	-0.019:0.026	0.78
**SPMS vs. PPMS**	0.0001	-0.024:0.025	0.97
**RRMS vs. PPMS**	-0.003	-0.029:0.024	0.84
**Group comparison**	**Longitudinal**		
	β	*CI*	*p*
**Rate of change in SPMS vs. RRMS**	-0.002	-0.005:0	<0.05
**Rate of change in SPMS vs. PPMS**	-0.002	-0.006:0.003	0.43
**Rate of change in RRMS vs. PPMS**	0.001	-0.003:0.004	0.76
**Network 11**			
**Group comparison**	**Cross-sectional**		
	β	*CI*	*p*
**SPMS vs. RRMS**	-0.018	-0.031:-0.006	<0.005*
**SPMS vs. PPMS**	-0.001	-0.015:0.012	0.87
**RRMS vs. PPMS**	0.017	0.002:0.032	<0.05
**Group comparison**	**Longitudinal**		
	β	*CI*	*p*
**Rate of change in SPMS vs. RRMS**	0.001	-0.001:0.002	0.43

**Rate of change in SPMS vs. PPMS**	-0.002	-0.005:0.001	0.22
**Rate of change in RRMS vs. PPMS**	-0.003	-0.006:0	0.07
**Network 12**			
**Group comparison**	**Cross-sectional**		
	β	*CI*	*p*
**SPMS vs. RRMS**	-0.031	-0.051:-0.012	<0.005*
**SPMS vs. PPMS**	-0.005	-0.026:0.016	0.63
**RRMS vs. PPMS**	0.026	0.003:0.049	<0.05
**Group comparison**	**Longitudinal**		
	β	*CI*	*p*
**Rate of change in SPMS vs. RRMS**	0.005	0.002:0.007	<0.0001*
**Rate of change in SPMS vs. PPMS**	0.004	-0.001:0.009	0.11
**Rate of change in RRMS vs. PPMS**	-0.001	-0.005:0.004	0.72
**Network 13**			
**Group comparison**	**Cross-sectional**		
	β	*CI*	*p*
**SPMS vs. RRMS**	-0.007	-0.027:0.013	0.47
**SPMS vs. PPMS**	-0.042	-0.064:-0.021	<0.0001*
**RRMS vs. PPMS**	-0.035	-0.059:-0.012	<0.005*
**Group comparison**	**Longitudinal**		
	β	*CI*	*p*
**Rate of change in SPMS vs. RRMS**	0.003	0.001:0.005	<0.005*
**Rate of change in SPMS vs. PPMS**	0.005	0.001:0.009	<0.05*
**Rate of change in RRMS vs. PPMS**	0.001	-0.002:0.005	0.43
**Network 14**			
**Group comparison**	**Cross-sectional**		
	β	*CI*	*p*
**SPMS vs. RRMS**	0.002	-0.028:0.031	0.92
**SPMS vs. PPMS**	0.002	-0.029:0.034	0.90

**RRMS vs. PPMS**	0.0001	-0.034:0.035	0.98
**Group comparison**	**Longitudinal**		
	β	*CI*	*p*
**Rate of change in SPMS vs. RRMS**	-0.005	-0.008:-0.002	<0.005*
**Rate of change in SPMS vs. PPMS**	-0.006	-0.013:0	<0.05
**Rate of change in RRMS vs. PPMS**	-0.001	-0.007:0.004	0.65
**Network 15**			
**Group comparison**	**Cross-sectional**		
	β	*CI*	*p*
**SPMS vs. RRMS**	-0.02	-0.032:-0.008	<0.001*
**SPMS vs. PPMS**	-0.01	-0.023:0.003	0.12
**RRMS vs. PPMS**	0.01	-0.004:0.024	0.16
**Group comparison**	**Longitudinal**		
	β	*CI*	*p*
**Rate of change in SPMS vs. RRMS**	0.001	0:0.003	0.07
**Rate of change in SPMS vs. PPMS**	0.005	0.003:0.008	<0.0001*
**Rate of change in RRMS vs. PPMS**	0.004	0.001:0.007	<0.005*
**Network 16**			
**Group comparison**	**Cross-sectional**		
	β	*CI*	*p*
**SPMS vs. RRMS**	-0.006	-0.018:0.007	0.38
**SPMS vs. PPMS**	-0.004	-0.017:0.01	0.59
**RRMS vs. PPMS**	0.002	-0.013:0.017	0.80
**Group comparison**	**Longitudinal**		
	β	*CI*	*p*
**Rate of change in SPMS vs. RRMS**	0.0001	-0.001:0.002	0.79
**Rate of change in SPMS vs. PPMS**	0.001	-0.002:0.004	0.47
**Rate of change in RRMS vs. PPMS**	0.001	-0.002:0.004	0.52
**Network 17**			

**Group comparison**	**Cross-sectional**		
	β	*CI*	*p*
**SPMS vs. RRMS**	-0.01	-0.031:0.012	0.37
**SPMS vs. PPMS**	-0.055	-0.078:-0.032	<0.0001*
**RRMS vs. PPMS**	-0.046	-0.071:-0.021	<0.0005*
**Group comparison**	**Longitudinal**		
	β	*CI*	*p*
**Rate of change in SPMS vs. RRMS**	0.008	0.005:0.011	<0.0001*
**Rate of change in SPMS vs. PPMS**	0.034	0.028:0.04	<0.0001*
**Rate of change in RRMS vs. PPMS**	0.025	0.02:0.031	<0.0001*
**Network 18**			
**Group comparison**	**Cross-sectional**		
	β	*CI*	*p*
**SPMS vs. RRMS**	-0.012	-0.025:0.001	0.06
**SPMS vs. PPMS**	-0.001	-0.015:0.013	0.88
**RRMS vs. PPMS**	0.011	-0.004:0.026	0.15
**Group comparison**	**Longitudinal**		
	β	*CI*	*p*
**Rate of change in SPMS vs. RRMS**	-0.001	-0.002:0.001	0.25
**Rate of change in SPMS vs. PPMS**	0.0001	-0.003:0.003	0.98
**Rate of change in RRMS vs. PPMS**	0.001	-0.002:0.003	0.50
**Network 19**			
**Group comparison**	**Cross-sectional**		
	β	*CI*	*p*
**SPMS vs. RRMS**	-0.005	-0.018:0.008	0.48
**SPMS vs. PPMS**	-0.002	-0.016:0.012	0.75
**RRMS vs. PPMS**	0.002	-0.013:0.018	0.76
**Group comparison**	**Longitudinal**		
	β	*CI*	*p*
**Rate of change in SPMS vs. RRMS**	-0.001	-0.003:0	0.053

**Rate of change in SPMS vs. PPMS**	-0.004	-0.007:-0.002	<0.005*
**Rate of change in RRMS vs. PPMS**	-0.003	-0.005:-0.001	<0.05
**Network 20**			
**Group comparison**	**Cross-sectional**		
	β	*CI*	*p*
**SPMS vs. RRMS**	-0.012	-0.028:0.005	0.16
**SPMS vs. PPMS**	-0.018	-0.036:-0.001	<0.05
**RRMS vs. PPMS**	-0.007	-0.026:0.012	0.48
**Group comparison**	**Longitudinal**		
	β	*CI*	*p*
**Rate of change in SPMS vs. RRMS**	0.001	-0.001:0.002	0.48
**Rate of change in SPMS vs. PPMS**	0.0001	-0.003:0.004	0.81
**Rate of change in RRMS vs. PPMS**	0.0001	-0.003:0.003	0.89
**Whole brain grey matter volume**			
**Group comparison**	**Cross-sectional**		
	β	*CI*	*p*
**SPMS vs. RRMS**	-0.012	-0.017:-0.006	<0.0001*
**SPMS vs. PPMS**	-0.003	-0.009:0.002	0.23
**RRMS vs. PPMS**	0.008	0.002:0.014	<0.01*
**Group comparison**	**Longitudinal**		
	β	*CI*	*p*
**Rate of change in SPMS vs. RRMS**	0.0001	-0.001:0.001	0.52
**Rate of change in SPMS vs. PPMS**	-0.001	-0.003:0.0001	0.13
**Rate of change in RRMS vs. PPMS**	-0.002	-0.003:0	<0.05

*Statistically significant after False Discovery Rate

Abbreviations: β = Beta coefficients; SPMS = Secondary Progressive Multiple Sclerosis; PPMS = Primary Progressive Multiple Sclerosis; RRMS = Relapsing Remitting Multiple Sclerosis; SE = Standard Error

Supplementary Table 6 Cross-sectional and longitudinal association of clinical disability (EDSS) with ICA GM networks and whole brain GM measures in training cohort.

		**Entire cohort** **(β, 95%CI, p)**
**Network 2**	**Baseline**	-0.003, -0.029:0.022, P=0.80
	**Rate of change**	-0.009, -0.016:-0.002, P<0.01*
**Network 3**	**Baseline**	-0.03, -0.070:0.010, P=0.14
	**Rate of change**	-0.011, -0.018:-0.004, P<0.005*
**Network 11**	**Baseline**	-0.017, -0.057:0.024, P=0.42
	**Rate of change**	-0.004, -0.011:0.004, P=0.31
**Network 12**	**Baseline**	-0.037, -0.071:-0.003, P<0.05
	**Rate of change**	-0.006, -0.014:0.001, P=0.09
**Network 13**	**Baseline**	-0.023, -0.058:0.012, P=0.19
	**Rate of change**	-0.015, -0.022:-0.007, P<0.0001*
**Network 14**	**Baseline**	-0.026, -0.056:0.005, P=0.10
	**Rate of change**	-0.009, -0.016:-0.001, P<0.05*
**Network 18**	**Baseline**	-0.016, -0.06:0.026, P=0.45
	**Rate of change**	-0.002, -0.010:0.005, P=0.53
**Network 19**	**Baseline**	-0.045, -0.081:-0.009, P<0.05
	**Rate of change**	-0.015, -0.022:-0.007, P<0.0001*
**GM volume**	**Baseline**	-0.18, -0.25:-0.11, P<0.0001*
	**Rate of change**	-0.009, -0.016:-0.001, P<0.05*

* Statistically significant after False Discovery Rate correction

Supplementary Table 7 Cross-sectional and longitudinal association of clinical disability (EDSS) with GM networks (obtained using Lasso) and whole brain GM measures in application cohort.

		Entire cohort (β, 95%CI, p)
**Network 2**	**Baseline**	-0.03, -0.071:0.011, P=0.16
	**Rate of change**	-0.024, -0.042:-0.006, P<0.01
**Network 3**	**Baseline**	-0.089, -0.157:-0.021, P<0.01*
	**Rate of change**	0.008, -0.01:0.026, P=0.38
**Network 11**	**Baseline**	-0.008, -0.076:0.059, P=0.81
	**Rate of change**	0.005, -0.012:0.023, P=0.55
**Network 12**	**Baseline**	-0.012, -0.064:0.04, P=0.65
	**Rate of change**	-0.019, -0.037:-0.001, P<0.05
**Network 13**	**Baseline**	-0.107, -0.157:-0.057, P<0.0001*
	**Rate of change**	0.008, -0.01:0.026, P=0.40
**Network 14**	**Baseline**	-0.014, -0.066:0.038, P=0.60
	**Rate of change**	0.004, -0.013:0.022, P=0.64
**Network 18**	**Baseline**	0.005, -0.06:0.069, P=0.89
	**Rate of change**	0.011, -0.007:0.028, P=0.23
**Network 19**	**Baseline**	-0.095, -0.154:-0.036, P<0.005*
	**Rate of change**	-0.001, -0.019:0.017, P=0.91
**GM volume**	**Baseline**	-0.162, -0.288:-0.037, P<0.05*
	**Rate of change**	0.001, -0.017:0.019, P=0.93

* Statistically significant after False Discovery Rate correction

Supplementary Table 8 Cross-sectional and longitudinal association of clinical disability (EDSS) with GM networks (obtained using ICA) and whole brain GM measures in application cohort.

		**Entire cohort (*β*, 95%CI, *p*)**
**Network 2**	**Baseline**	-0.011, -0.065:0.044, P=0.71
	**Rate of change**	0.001, -0.018:0.018, P=0.99
**Network 3**	**Baseline**	-0.013, -0.079:0.053, P=0.69
	**Rate of change**	0.005, -0.013:0.023, P=0.60
**Network 11**	**Baseline**	-0.047, -0.094:0.001, P=0.054
	**Rate of change**	0.022, 0.004:0.04, P<0.05
**Network 12**	**Baseline**	-0.089, -0.151:-0.027, P<0.005*
	**Rate of change**	0.007, -0.01:0.025, P=0.41
**Network 13**	**Baseline**	0.013, -0.057:0.083, P=0.71
	**Rate of change**	-0.001, -0.019:0.017, P=0.93
**Network 14**	**Baseline**	0.003, -0.048:0.053, P=0.92
	**Rate of change**	-0.017, -0.035:0.001, P=0.06
**Network 18**	**Baseline**	0.005, -0.061:0.071, P=0.88
	**Rate of change**	0.012, -0.005:0.03, P=0.18
**Network 19**	**Baseline**	-0.066, -0.123:-0.01, P<0.05*
	**Rate of change**	0.001, -0.016:0.019, P= 0.87
**GM volume**	**Baseline**	-0.162, -0.288:-0.037, P<0.05*
	**Rate of change**	0.001, -0.017:0.019, P=0.93

*Statistically significant after False Discovery Rate correction

Supplementary Table 9 Assessing treatment effect using GM network measures obtained using Lasso.

	Network 2			
	% change	β	95% CI	p
**Annual rate of change**				
Treatment group	1.03	-0.010	-0.015:-0.005	<0.0001*
Placebo group	2.17	-0.012	-0.019:-0.004	<0.005*

	Network 3			
	% change	β	95% CI	p
**Annual rate of change**				
Treatment group	0.46	-0.005	-0.005:-0.004	<0.0001*
Placebo group	0.70	-0.003	-0.004:-0.001	<0.0001*

	Network 11			
	% change	β	95% CI	p
**Annual rate of change**				
Treatment group	0.66	-0.007	-0.008:-0.006	<0.0001*
Placebo group	0.88	-0.002	-0.004:-0.001	<0.001*

	Network 12			
	% change	β	95% CI	p
**Annual rate of change**				
Treatment group	1.23	-0.012	-0.014:-0.011	<0.0001*
Placebo group	1.58	-0.004	-0.006:-0.001	<0.005*

	Network 13			
	% change	β	95% CI	p
**Annual rate of change**				
Treatment group	0.29	-0.003	-0.004:-0.001	<0.0001*
Placebo group	0.82	-0.005	-0.008:-0.003	<0.0001*

	Network 18			
	% change	β	95% CI	p
**Annual rate of change**				
Treatment group	0.52	-0.005	-0.006:-0.004	<0.0001*
Placebo group	0.75	-0.002	-0.004:-0.001	<0.005*

	Network 19			
	% change	β	95% CI	p
**Annual rate of change**				

	% change	β	95% CI	p
Treatment group	0.60	-0.006	-0.007:-0.005	<0.0001*
Placebo group	0.83	-0.002	-0.004:-0.001	<0.05*

	**Network 20**			
	% change	β	95% CI	p
**Annual rate of change**				
Treatment group	0.99	-0.010	-0.011:-0.009	<0.0001*
Placebo group	1.29	-0.003	-0.005:-0.001	<0.005*

	**Grey matter**			
	% change	β	95% CI	p
**Annual rate of change**				
Treatment group	0.49	-0.005	-0.005:-0.005	<0.0001*
Placebo group	0.63	-0.001	-0.020:-0.001	<0.0001*

***Statistically significant after False Discovery Rate (FDR) correction**

The table shows the results of mixed effect models where the interaction between time and treatment was the fixed (explanatory) effect and network loadings were the outcome measure. Eight networks and whole brain GM volume showed a treatment effect: participants in the treated arm had a lower rate of volume change (higher content of GM) over time than those in the placebo group.

Abbreviations: β = Beta Estimates

Supplementary Table 10 Assessing treatment effect using GM network measures obtained using ICA fitted on the application cohort.

	**Network 3**			
	% change	β	95% CI	p
**Annual rate of change**				
Treatment group	0.76	-0.008	-0.009:-0.007	<0.0001*
Placebo group	0.95	-0.002	-0.003:-0.001	<0.01*

	**Network 4**			
	% change	β	95% CI	p
**Annual rate of change**				
Treatment group	0.54	-0.005	-0.006:-0.005	<0.0001*
Placebo group	0.82	-0.003	-0.004:-0.001	<0.0001*

	**Network 5**			
	% change	β	95% CI	p
**Annual rate of change**				
Treatment group	0.56	-0.006	-0.007:-0.005	<0.0001*
Placebo group	1.05	-0.005	-0.007:-0.003	<0.0001*

	**Network 6**			
	% change	β	95% CI	p
**Annual rate of change**				
Treatment group	0.81	-0.008	-0.010:-0.007	**<0.001***
Placebo group	0.51	0.003	0.001:0.005	**<0.05***

	**Network 12**			
	% change	β	95% CI	p
**Annual rate of change**				
Treatment group	0.56	-0.006	-0.007:-0.005	<0.0001*
Placebo group	0.76	-0.002	-0.003:-0.001	<0.005*

	**Network 13**			
	% change	β	95% CI	p
**Annual rate of change**				
Treatment group	0.76	-0.008	-0.009:-0.007	<0.0001*
Placebo group	0.91	-0.002	-0.003:-0.0002	<0.05*

	**Network 14**			
	% change	β	95% CI	p
**Annual rate of change**				
Treatment group	1.47	-0.015	-0.017:-0.013	<0.0001*
Placebo group	1.93	-0.005	-0.007:-0.002	<0.001*

	Network 17			
	% change	β	95% CI	p
**Annual rate of change**				
Treatment group	0.55	-0.006	-0.009:-0.002	<0.005*
Placebo group	1.45	-0.009	-0.015:-0.004	<0.001*

	Network 18			
	% change	β	95% CI	p
**Annual rate of change**				
Treatment group	0.48	-0.005	-0.006:-0.004	<0.0001*
Placebo group	0.72	-0.002	-0.004:-0.001	<0.0005*

	Network 20			
	% change	β	95% CI	p
**Annual rate of change**				
Treatment group	0.91	-0.009	-0.011:-0.008	<0.0001*
Placebo group	1.26	-0.004	-0.006:-0.001	<0.001*

	Grey matter			
	% change	β	95% CI	p
**Annual rate of change**				
Treatment group	0.49	-0.005	-0.005:-0.005	<0.0001*
Placebo group	0.63	-0.001	-0.002:-0.001	<0.0001*

***Statistically significant after False Discovery Rate (FDR) correction**

The table shows the results of mixed effect models where the interaction between time and treatment was the fixed (explanatory) effect and network loadings were the outcome measure. Eight networks and whole brain GM volume showed a treatment effect: participants in the treated arm had a lower rate of volume change (higher content of GM) over time than those in the placebo group.

Abbreviations: β = Beta Estimates

Supplementary Table 11 Sample size estimation for each arm for treatment effect detection with a significance level of 0.05 and a power of 0.80

	**Sample size**
**Network 2**	526
**Network 3**	550
**Network 11**	477
**Network 12**	565
**Network 13**	227
**Network 18**	756
**Network 19**	763
**Network 20**	1684
**Whole brain GM volume**	753

Supplementary Figure 1. Rate of grey matter volume loss across multiple sclerosis phenotypes over time.

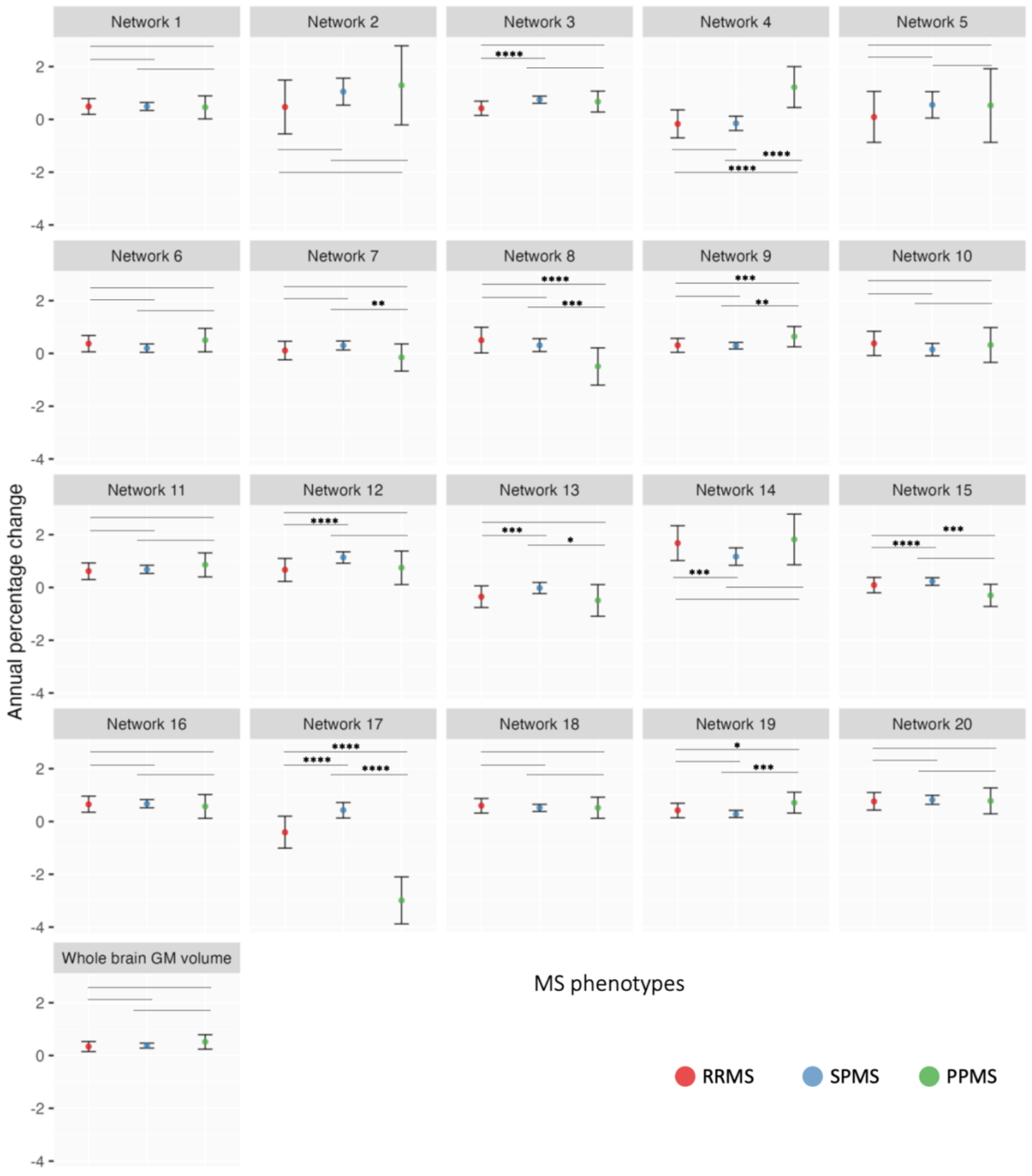

Caption. The rate of change in grey matter networks differed among MS phenotypes over time. Network 3 and 12 differed between participants with SPMS and RRMS, showing a greater

annual percentage change in network loading (more GM atrophy) in people with SPMS than in RRMS. Networks 4, 9, and 19 over time differed between people with PPMS and those with RRMS and SPMS, with a faster volume loss rate in people with PPMS. Network 7 differed between participants with SPMS and PPMS, with faster volume loss in people with SPMS. Network 8 over time differed between people with PPMS and those with RRMS and SPMS, with a higher rate of volume change in RRMS and SPMS. Networks 13 and 17 differed between participants with SPMS and those with RRMS and PPMS, with a faster volume loss rate in people with SPMS. Network 14 showed a faster rate of volume change in people with RRMS than in SPMS, and network 17 a faster volume loss in participants with RRMS than in those with PPMS. Whole brain GM volume did not differ among MS phenotypes after correcting for multiple comparisons.

**** P<0.0001; *** P<0.005; ** P<0.01; *P<0.05

Legend: GM, grey matter; MS, multiple sclerosis; RRMS, relapsing remitting multiple sclerosis; SPMS, secondary progressive multiple sclerosis; PPMS, primary progressive multiple sclerosis
